# Bi-layer photonic random meta-composite for cryogenic thermal control by ultra-broadband scattering matched reflectance

**DOI:** 10.1038/s41377-026-02372-9

**Published:** 2026-06-30

**Authors:** Hongchao Li, Hexiang Han, Zhiyuan Zhao, Hao Gong, Xiaokun Song, Zhongyang Wang, Gang Liu, Tongxiang Fan, Xiao Zhou, Di Zhang

**Affiliations:** 1 State Key Laboratory of Metal Matrix Composites, School of Materials Science and Engineering, Shanghai Jiao Tong University, Shanghai, 200240 China; 2 Shanghai Institute of Spacecraft Equipment, Shanghai, 200240 China

**Keywords:** Metamaterials, Nanoparticles, Solar energy and photovoltaic technology, Mid-infrared photonics

## Abstract

Cryogenic thermal control coatings represent a significant advancement over existing coatings limited to high reflectance in the 0.2–2.5 μm solar spectrum range, being able to reflect 96.6% of solar irradiance and to reach about 145 K equilibrium temperature, offering transformative potential for deep-space exploration, remote sensing, and other cryogenic applications. To achieve the cryogenic temperature further lower than 100 K in space, the peculiar optical property to reflect 99.9% of solar irradiance becomes necessary, which can be realized by an ultra-broadband reflectance from 0.2 to 8 μm. Here, we propose a bi-layer meta-composite comprising two distinct photonic random media, where rationally designed scatterer sizes selectively target short- and long-wavelength solar irradiance. By matching the scattering peak regimes, the meta-composite achieves a weighted solar reflectance of 97.3% over an ultrabroad 0.2–8 μm region. In the home-built deep-space simulator, the bi-layer meta-composite maintains an equilibrium temperature of 145 K, superior to existing coatings designed for room-temperature thermal control. Ground simulated irradiation tests further demonstrate exceptional optical stability under charged particles and atomic oxygen irradiations, with degradation of less than 1.5%. Moreover, the meta-composite retains optical properties comparable to existing coatings even after long-term ultraviolet irradiation. This work not only highlights the viability of photonic random meta-composites for cryogenic thermal control but also introduces a scattering regime matching strategy to broaden the spectral selectivity of disordered photonic systems.

## Abstract (web summary)

A bi-layer meta-composite enables cryogenic space thermal control via hierarchical scattering in an ultra-broadband wavelength range.

## Graphical abstract

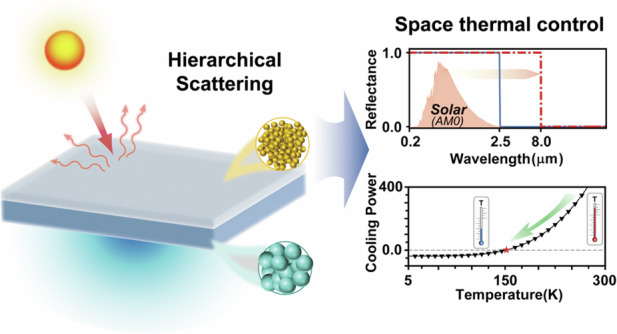

## Introduction

Cryogenic technology has emerged as a critical enabler for humanity’s expanding space endeavors, from addressing fundamental questions in space science to facilitating deep-space exploration and expanding a human presence across the solar system^1–3^. How to enable heat dissipation to maintain cryogenic thermal control is of great importance to cryogenic technology and space objects. However, it is still difficult to maintain stable cryogenic thermal control due to the scenario that thermal radiation is the sole way for heat dissipation in the space environment^4–6^. Among various strategies for cryogenic thermal control, the thermal control coating has been considered as an effective way to cool spacecraft exposed to solar irradiance^7^. Taking advantage of the spectrally selective response, thermal control coatings provide cooling through minimizing or eliminating the absorption of solar irradiance and emitting thermal radiation for heat dissipation^8–11^. However, existing thermal control coatings fail to attain the required cryogenic temperatures due to their excessive solar absorptance, which remains a significant challenge.

**Fig. 1:**
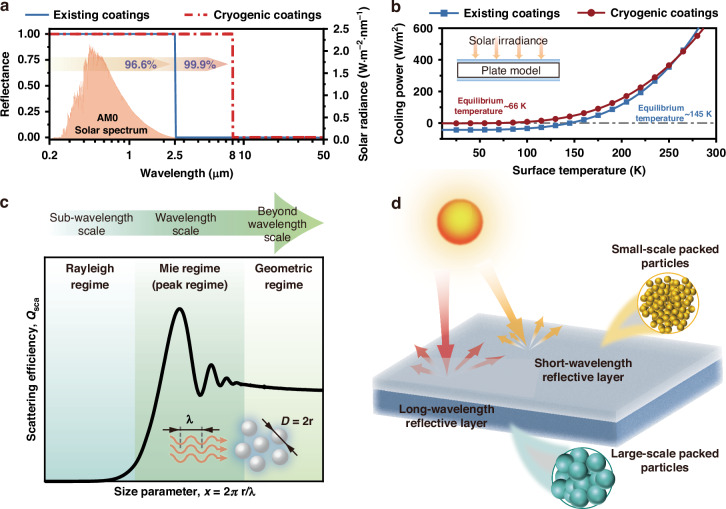
Principle of the bi-layer meta-composite for space cryogenic thermal control. **a** Reflectance spectra of ideal existing thermal control coatings and cryogenic coatings; **b** Theoretical space cooling powers and equilibrium temperatures of two different coatings; The inset is the plate model coated on both sides and perpendicular to the solar used for equilibrium temperature calculations; **c** Scattering regime dependence on the relationship between wavelength and characteristic sizes of particles; **d** Schematic image of bi-layer photonic random media meta-composite

The above-mentioned bottleneck regarding high solar absorptance in existing thermal control coatings mainly originates from the unsatisfied spectrum-dependent reflectance property. In reality, most existing thermal control coatings focus on the improvement of solar reflectance at a relatively narrow 0.2–2.5 μm solar spectrum (see Fig. 1a). However, such spectrum design strategy that ideally realizes the unit reflectance within 0.2–2.5 μm can only make the coating reject about 96.6% of solar irradiance, and the unit emittance beyond 2.5 μm covers almost all radiation of the ideal blackbody at 300 K. Based on this design, this ideal coating can only reach about 145 K theoretical equilibrium temperature in space (see “Materials and methods” section for more details about the equilibrium temperature and cooling power), which is still not low enough for space cryogenic applications, as shown in Fig. 1b. In the 1960s, Hibbard demonstrated theoretically that the equilibrium temperature of an ideal coating in space can reach the cryogenic temperature lower than 100 K if the coating can ideally reflect solar irradiance for ultrabroad 0.2–8 μm region and emit strongly beyond this region^12^, as shown in Fig. 1a. Figure 1a compares the ideal reflectance spectrum of this cryogenic coating derived from Hibbard’s theory to that of the ideal existing coating. The ideal cryogenic coating is capable of reflecting about 99.9% of solar irradiance. The variation in the transition wavelength from 2.5 μm to 8 μm shrinks the high emittance region, and the thermal dissipation through radiation weakens. However, the blackbody radiation at 100 K is about 3 orders of magnitude lower than that at 300 K and also significantly lower than the solar irradiance, as shown in Fig. S1. This indicates that reflecting the solar irradiance is more vital for cryogenic thermal control than enhancing the emittance of thermal radiation. In Fig. 1b, this ideal cryogenic coating is able to make the temperature of both sides coated plate drop to 66 K, much lower than the existing coatings due to the lower absorption of solar irradiance. According to Hibbard’s theory, the equilibrium temperature decreases with the red shift of the transition wavelength due to the expansion of the reflective band. However, only 0.1% of the solar irradiance lies beyond 8 μm, and further broadening the reflective band has a negligible effect. Moreover, most materials are unable to keep non-absorbing from 0.2 μm at least to the mid-infrared region. Therefore, a transition wavelength at 8 μm represents a more practical solution to current engineering challenges for thermal control coatings.

Despite the novel strategy for cryogenic thermal control in space by Hibbard’s theory, there still exists an unsolved issue regarding how to achieve the high reflectance in the ultrabroad region from 0.2 μm to 8 μm. Current advances in thermal control coatings mainly concentrate on the improvement of reflectance in the 0.2–2.5 μm spectrum range^13,14^. Among these, the photonic random media (PRM) leveraging strong scattering have attracted considerable attention due to their low cost and scalability^15^. For instance, the hollow microspheres with varying refractive index and extinction coefficient have been studied to enhance the solar reflectance in the 0.2–2.5 μm region^16^; random polyimide nanofiber films have been optimized at the microscale level for high solar reflectance^17^; silica aerogels with a hybrid structure consisting of nanofibers and microspheres can reach 99.1% solar reflectance within 0.2–2.5 μm^4^. However, exploration of coatings targeting ultra-broadband (i.e., 0.2–8 μm) reflectance remains scarce. Youngquist et al. proposed a theoretical selective surface model combining a paint-like scattering top layer with a silver mirror substrate^18–20^, yet practical applications are still limited due to the quality of the interface between scattering layers/metallic reflectors and the strong dependence on the underlying substrate. Such limitations constrain the optical properties of the proposed structure, resulting in inadequate solar reflectance for cryogenic requirements. This persistent performance gap underscores the urgent need for innovative coatings capable of achieving near-unity reflectance across the 0.2–8 μm region.

These unsolved challenges motivate the development of new strategies for ultra-broadband reflective microstructures design. As shown in Fig. 1c, the scattering efficiency (*Q*_sca_, the unitless parameter defined as the ratio of the scattering cross-section and the geometric cross-section) of non-absorbing particles can be approximately divided into three different regimes by size parameters (*x* = 2*πr*/*λ, r* the radius of the particle and *λ* the incident wavelength)^21,22^: (1) The Rayleigh regime where the characteristic scale is sub-wavelength (*x* « 1) and the multiple interactions between photons and scatterers are limited; (2) The Mie regime or peak regime, where the *Q*_sca_ is obviously enhanced and shows peak with the scatterers reaching the wavelength scale (*x* ≈ 1); (3) The geometric regime (*x* » 1) where the *Q*_sca_ is almost independent of *x* and asymptotic to the geometric limit of 2. For mono-layer PRM, the reflective band is constrained within 2.5 μm due to the mismatch of the scatterers in each scattering regime. The scattering of sub-micron scatterers in existing mono-layer PRM is dominated by the Mie regime in visible region of solar spectrum but suffer from low Rayleigh scattering efficiency in the mid-infrared spectrum region. Such scenario thus results in the unsatisfied reflectance in the 2.5–8 μm range. Considering this limitation, we adopted hierarchical PRM to reach scattering regimes matching, which enables effective solar reflectance within the ultra-broadband (namely 0.2–8 μm) through the piece-wise spectral response. According to Fig. 1c, it is possible to match the interested wavelength band and the peak regime by varying the characteristic scale of scatterers. Such hierarchical strategy offers an intriguing design principle for the meta-composite with hierarchical PRM to tune light-matter interactions in different regions^23^.

The emerging bi-layer PRM presents a promising way to expand the design space for performance optimization of particulate media. Some studies have leveraged this architecture to achieve multi-functionality^24,25^, such as constituting a colored and solar-infrared reflective coating with a visible-absorptive paint atop^26^ or imparting super-hydrophobicity via incorporating hydrophobic modifiers into the upper particulate medium^27^. More importantly, and more instructive to this work, the bi-layer structure could improve the spectral performance of PRM by tuning scattering regimes to enhance the scattering efficiency across different wavebands^25,27,28^. This is exemplified by the use of a SiO_2_ particle masking layer to raise the ultraviolet (UV) reflectance of a TiO_2_ under-layer above 85%^27^, demonstrating effective scattering regulation across specific bands. Nevertheless, existing research on such bi-layer PRM remains confined to the near-infrared spectrum (up to 2.5 μm). Extending this architecture design to achieve ultra-broadband reflectance into the mid-infrared region (up to 8 μm) still needs further investigation.

Based on the proposed scattering regime matching principle, in this work, we prepared a bi-layer meta-composite consisting of hierarchical PRM, as schematically displayed in Fig. 1d. The characteristic sizes of scatterers in upper and lower PRM were designed rationally to respond to the short- and long-wavelength solar irradiance, respectively. And the weighted solar reflectance from 0.2 μm to 8 μm reaches 97.3% due to the hierarchical scattering peak regime. The equilibrium temperature of this hierarchical PRM meta-composite was down to only 145 K, tested experimentally by a home-built deep-space simulator, lower than the existing thermal control coatings obviously. It is worthy to note that the fabricated hierarchical PRM meta-composite is all-inorganic and shows excellent resistance to simulated space environment, including charged particles, atomic oxygen, and UV irradiation. Overall, we suggest that the hierarchical scattering design principle offers a new strategy for PRM to expand the range of spectrally selective responses. And the undeniable potential of hierarchical PRM meta-composite is verified to achieve the cryogenic thermal control, which is critical for practical deployments.

## Results

### The structure and optical properties of a bi-layer photonic random meta-composite

**Fig. 2:**
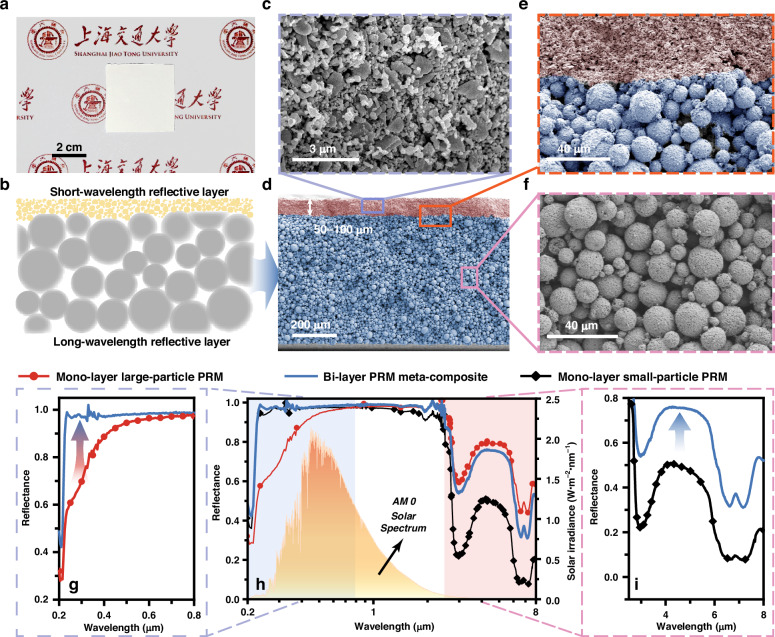
Structure and optical properties of the bi-layer meta-composite. **a** Optical image of the bi-layer PRM meta-composite; **b** Two-dimensional schematic image of the bi-layer PRM meta-composite; SEM images of the upper PRM layer composed of small-scale particles (**c**), the cross-section of the bi-layer PRM meta-composite (**d**), the interface between upper and lower layers (**e**), and the lower PRM layer composed of large-scale particles (**f**); **g**–**i** Comparisons of reflectance corresponding to mono-layer and bi-layer PRM

Recently, ever-increasing attention has been paid to the PRM composed of dielectric scatterers with certain structural parameters and random spatial coordinates. Such objects have been demonstrated to strongly reflect the broadband solar irradiance based on the light scattering, balancing the advantages of low cost and large-scale processing^29–31^. The hierarchical PRM meta-composite in this work consists of two layers of such typical PRM with different structural parameters. The PRM with various characteristic sizes can be sequentially stacked by tape-casting layer by layer (see Fig. S2 in Supplementary Information (SI)). Taking advantage of the tape-casting method, the bi-layer PRM meta-composite can be easily processed into arbitrary shapes^32^. And the optical image of a 4 cm × 4 cm bi-layer PRM meta-composite shaped into a square is exhibited in Fig. 2a (scale bar: 2 cm).

Figure 2b illustrates the bi-layer hierarchical meta-composite, consisting of an upper layer of randomly packed Y_2_O_3_ particles with small-scale characteristic sizes and a lower layer of randomly packed Y_2_O_3_ microspheres with large-scale characteristic sizes. This meta-composite was fabricated using a temporary organic dispersant and binder (see “Materials and methods” section for details), which were subsequently removed through post-sintering at 600 °C, resulting in a binder-free structure. The lower PRM is much thicker than the upper PRM, giving rise to more intensive light-particle scattering and the avoidance of photon transmission. The cross-section characterized by the scanning electron microscope (SEM) (Fig. 2d, scale bar: 200 μm) confirms the bi-layer hierarchical design in Fig. 2b. According to previous reports^33,34^, the size distributions of polydisperse particles can be characterized well by two structural parameters, namely, effective radius (*r*_eff_) and effective variation (*v*_eff_), which are defined with the consideration of scattering characteristics (see the detailed definition in SI). Therefore, *r*_eff_ and *v*_eff_ were adopted to describe the size distributions of Y_2_O_3_ particles in our bi-layer meta-composite. The upper PRM layer with a thickness of 50–100 μm contains closely packed, irregular Y_2_O_3_ particles with a *r*_eff_ of 0.7, a *v*_eff_ of 0.3, and a volume fraction (*f*_v_) of 0.55 (Fig. 2c, scale bar: 3 μm). The Y_2_O_3_ particles in the lower PRM layer are obviously larger and more regular as displayed in Fig. 2f (scale bar: 40 μm), whose characteristic sizes are *r*_eff_ = 8, *v*_eff_ = 0.3, and *f*_v_ = 0.50. Both Y_2_O_3_ scatterers exhibit log-normal size distributions, as confirmed in Fig. S3. And the volume fractions of the two layers in the hierarchical meta-composite were independently verified through the Archimedes method and mercury intrusion porosimetry (MIP) measurements^35,36^ (Fig. S4). Although the final bi-layer meta-composite consists of Y_2_O_3_ particles without other materials, these particles are not simply packed together. After removal of the additives at 600 °C, the 1200 °C sintering was carried out to facilitate the formation of necks between particles and to ensure sufficient mechanical properties of the bi-layer meta-composite (see more details in “Materials and methods” section and SI). Due to the difference in characteristic sizes, the upper and lower PRM are separated obviously, and the interface between these two layers in Fig. 2e is clear (scale bar: 40 μm). The interface without binders, though topologically rough and discontinuous, allows continuous light transfer in the bi-layer hierarchical structure without boundary interference. And the mechanical integrity of the bi-layer meta-composite has been demonstrated by Vickers microhardness tests, despite interfacial roughness (Fig. S7).

The bi-layer hierarchical PRM meta-composite achieves 97.3% weighted solar reflectance ($\bar{R}$) across 0.2–8 μm (Fig. 2h), which is calculated via AM0 solar irradiance spectrum. Compared to the mono-layer PRM with only the large-scale scatterers, the bi-layer meta-composite overcomes the deficiency of reflectance degradation, as shown in Fig. 2g when the thickness is kept constant. This enhancement is particularly pronounced within the 0.2–2.5 μm range, where most solar irradiance is concentrated. The sharp decrease of reflectance around 0.23 μm results from the intrinsic absorption of Y_2_O_3_ caused by the bandgap transition (the bandgap of Y_2_O_3_ is 5.6 eV^37^). In contrast to the silver reflector that exhibits strong intrinsic absorption below 0.4 μm, the bi-layer PRM meta-composite effectively reduces UV irradiance absorptance by about 8% (Fig. S8). This result highlights the superiority of the PRM over conventional second-surface mirrors. In the mid-infrared region, typically from 2.5 μm to 8 μm, photons interact weakly with the small-scale scatterers in the upper PRM, which transmit to the lower PRM and continue to interact with large-scale scatterers. Such behavior could result in a 20–30% increase in the reflectance of bi-layer meta-composite compared to the mono-layer PRM with only the small-scale scatterers and similar thickness (Fig. 2i). The comparison of reflectance spectra in the 0.2–8 μm range (Fig. 2h) clearly exhibits that the bi-layer PRM meta-composite integrates the spectral advantages of two mono-layer PRM. In addition, there exist absorptance peaks around 3 μm and 7 μm due to the intrinsic absorption of –OH and –OC bonds^38^. Since Y_2_O_3_ is hygroscopic, scatterers tend to absorb moisture and carbon dioxide in the air, which has been proved by the X-ray photoelectron spectroscopy (XPS) spectrum as displayed in Fig. S9.

### The mechanism of scattering regime matching

The bi-layer PRM meta-composite achieves the high reflectance in the broadband region (0.2–8 μm) through its hierarchical structure design based on scattering regimes matching. To reveal the underlying mechanism, we investigated the optical properties and basic scattering characteristics of an individual PRM layer.

**Fig. 3:**
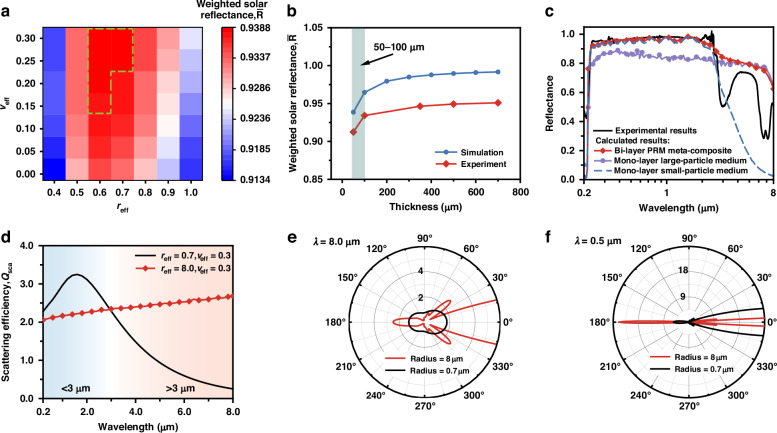
Mechanism of scattering regime matching for ultra-broadband reflectance. **a** Dependence of weighted solar reflectance on *r*_eff_ and *v*_eff_; **b** The weighted solar reflectance of the upper PRM layer with different thickness; **c** The reflectance spectra of mono-layer and bi-layer PRM; **d** Scattering efficiency, *Q*_sca_, of particles with *r*_eff_ = 0.7, *v*_eff_ = 0.3 and *r*_eff_ = 8.0, *v*_eff_ = 0.3; **e** Phase function of particles with radius = 0.7 μm and 8 μm at wavelength *λ* = 8 μm; **f** Phase function of particles with radius = 0.7 μm and 8 μm at wavelength *λ* = 0.5 μm

Tuning the scatterers’ size distribution by defined structure parameters *r*_eff_ and *v*_eff_ (see the definition in SI), we predicted the range of scatterer size that designed the best optical properties by the double optimized Monte Carlo simulations (MC)^33^. As representatively shown in Fig. 3a, the Y_2_O_3_ particles with structural parameters *r*_eff_ and *v*_eff_ falling within the dashed boundary achieve optimal weighted solar reflectance within 0.2–8 μm. The predictive volume fraction in double optimized MC is 55%, which is consistent with the experimental results (see more details in SI) and typical for binder-free stacked structures^39^. Such a prediction offers an aid to a priori-determine the Y_2_O_3_ particles in upper PRM with *r*_eff_ = 0.7 and *v*_eff_ = 0.3. The investigation of thickness-dependent performance reveals that the weighted solar reflectance of small-particle PRM increases progressively with thickness varying from 50 μm to 700 μm (Fig. 3b, and full reflectance spectra are included in Fig. S10). Calculated results obtained by our double optimized MC method show a similar trend to the experimental data, with the greatest variation between 50 μm and 100 μm. When the thickness is larger than 100 μm, the weighted solar reflectance increases slowly. This result proves the rationality of the thickness control for the upper PRM in Fig. 2d.

The 50-μm-thick upper PRM with scatterers distribution of *r*_eff_ = 0.7 and *v*_eff_ = 0.3 maintains the reflectance higher than 90% in the vicinity of 0.2–2.5 μm, while the spectral reflectance decreases rapidly beyond 2.5 μm (see Fig. 3c). This behavior arises from the fundamental transition between scattering regimes, as evidenced by the scattering efficiency profile of the particle cloud with the same distribution (Fig. 3d). According to the relationship between characteristic sizes and wavelength, the Mie scattering regime is dominant within 0.2–2.5 μm, which corresponds to the region where solar irradiance concentrated, followed by the Rayleigh scattering when the wavelength band extends into 2.5–8 μm. The characteristic sizes are located in the sub-wavelength scale, and the photon-scatterer interactions get weak^40,41^. On the contrary, scatterers with the distribution of *r*_eff_ = 8 and *v*_eff_ = 0.3 own the characteristic sizes of the wavelength scale, even the beyond-wavelength scale, which correspond to the Mie regime and geometric regime within 2.5–8 μm. The interactions between these large-scale scatterers and the mid-infrared wavelength could be much stronger. The 500-μm-thick PRM comprised of large-scale scatterers, was simulated by the MC method based on the geometric optics (see SI for the computational flowcharts and the verification)^42,43^. It exhibited high reflectance beyond 0.2–2.5 μm as we expected. And the *Q*_sca_ of scatterers with *r*_eff_ = 8, *v*_eff_ = 0.3 shows stronger scattering over the 2.5–8 μm than the small-scale scatterers (Fig. 3d), which means the interested mid-infrared region matches with the Mie regime, even the geometric region, instead of the Rayleigh regime.

To visualize the different scattering regimes, we display the scattering phase functions in Fig. 3e and f for two scatterer sizes at representative 8 μm and 0.5 μm, respectively. At the wavelength of 8 μm, the scatterers with a radius of 0.7 μm are much smaller than the wavelength, resulting in an equivalent forward versus backward scattering phase function. This typical phase function of Rayleigh scattering shows a poor diffraction effect. In contrast, the scatterers with a radius of 8 μm produce the well-defined Mie scattering characteristics, where the scatterers generate more significant forward scattering and diffraction patterns than the Rayleigh scattering. Although the angular distribution of Rayleigh scattering is more uniform, the weaker scattering efficiency is undesired for mid-infrared reflectance. At the wavelength of 0.5 μm, the 0.7 μm scatterers change to the Mie scattering regime as the characteristic size is comparable to the wavelength of light. Furthermore, the geometric scattering, dominated by the strongly peaked forward and backward scattering in the narrow regions, is observed clearly when the light is incident on the 8 μm scatterers. Both the Mie scattering and geometric scattering predominately have the forward scattering phase function, the scattering directions in Mie scattering, however, are more diverse to deflect the path of the light transfer^41^.

The above analysis of scattering efficiency and phase functions suggests that the large-scale scatterers (*r*_eff_ = 8, *v*_eff_ = 0.3) in the lower PRM layer, operating in the Mie and geometric scattering regimes, effectively compensate for the limited scattering performance of small-scale Rayleigh scatterers (*r*_eff_ = 0.7, *v*_eff_ = 0.3) in the upper PRM layer. We also investigated the influence of different size distributions (Fig. S14a) on this complementary scattering phenomenon. Due to the dominant geometric scattering regime, further increase of the particle size fails to enhance the scattering efficiency in the lower PRM layer (Fig. S14b). In contrast, larger particles exhibit reduced number density at a fixed volume fraction (Fig. S14c), which ultimately reduces the light-scattering frequency of particles in the lower PRM. Further analysis indicates that although the scattering efficiencies of particles with intermediate sizes (*r*_eff_ = 3, *v*_eff_ = 0.3 and *r*_eff_ = 5, *v*_eff_ = 0.3) are higher than our selected scatterers with *r*_eff_ = 8, *v*_eff_ = 0.3 in lower PRM layer (Fig. S14b), their larger specific surface area (Fig. S14d), resulting from the predominance of smaller particles in the polydisperse system, would exacerbate moisture absorption in the hygroscopic Y_2_O_3_ particles. These comprehensive considerations guided our final selection of *r*_eff_ = 8, *v*_eff_ = 0.3 particles for the lower PRM layer.

Then, we compared the experimental and calculated reflectance spectra to validate the complementary scattering phenomena between hierarchical PRM layers. Taking the bi-layer hierarchical meta-composite composed of 50-μm-thick upper PRM and 500-μm-thick lower PRM as an example, we tested the reflectance spectrum experimentally within 0.2–8 μm (the black line in Fig. 3c). Meanwhile, the modified MC method, combining the double optimized MC method with the geometric optics MC method, was carried out to simulate the spectrum of the bi-layer meta-composite with the same structural parameters as the tested sample. The modification of the MC method here ensures that photons can transfer between the upper and lower PRM continuously. Taking the scattering characteristics of two sizes of scatterers as inputs, the calculated results are consistent with the experimental results well (Fig. 3c). Such consistency confirms that the bi-layer PRM meta-composite achieves excellent optical properties originating from the combination of the strong scattering performance of two sizes of scatterers in different wavelength bands due to the match of the scattering regime.

### The thermal control performance in the space simulator

**Fig. 4:**
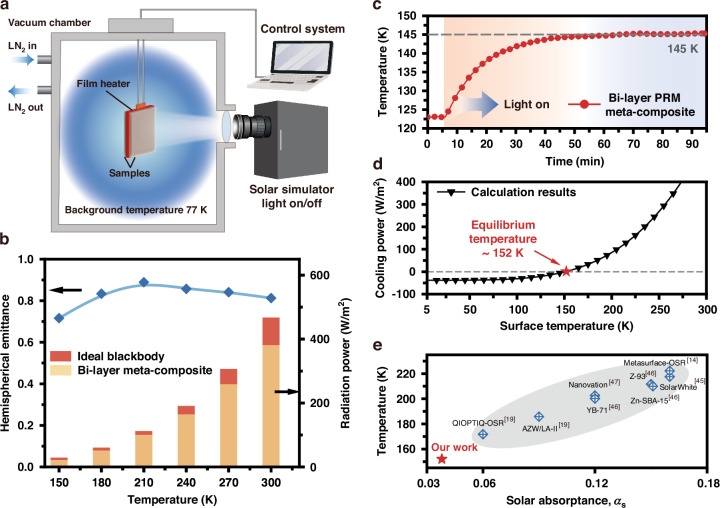
Cryogenic thermal control performance of the bi-layer meta-composite. **a** Schematic image of the home-built space cryogenic simulator; **b** Hemispherical emittance and thermal radiation power under different temperatures tested by the steady-state calorimetric method; **c** Temperature of the bi-layer PRM meta-composite after the AM0 solar simulator was turned on; **d** Theoretical relationship between cooling power and temperature of the bi-layer PRM meta-composite; **e** Equilibrium temperature and solar absorptance of current thermal control coatings and the bi-layer meta-composite

In addition to the evaluation of reflectance and the underlying mechanism of the bi-layer PRM meta-composite, here we further investigated the thermal control performance in the space simulator. The dual-function space simulator, schematically shown in Fig. 4a, was built for lab-scale experiments. By controlling the switch of light, it could be used to characterize temperature-dependent emittance and monitor the real-time temperature variance of coatings.

The hemispherical emittance of the bi-layer PRM meta-composite was characterized at different temperatures by the steady-state calorimetric technique with the light kept off^44^. According to the thermal equilibrium principle, the thermal radiation power of the samples is equal to the heating power of the electric-driven film heater when the sample temperature is fixed as a constant. With the background temperature at 77 K, the sample temperature (*T*) was set to 150 K, 180 K, 210 K, 240 K, 270 K, and 300 K, respectively, and the heating powers of the film heater were recorded as the radiation power of the sample after reaching the steady state. The hemispherical emittance of the sample is defined as the ratio of the radiation power to that of the ideal blackbody. In Fig. 4b, the hemispherical emittance of the bi-layer meta-composite increases first and then decreases with the temperature dropping from 300 K to 150 K. The minimum of the hemispherical emittance is nearly 0.7 at 150 K. The histogram in Fig. 4b, exhibiting the radiation powers of the bi-layer meta-composite and the ideal blackbody, reveals that the influence of the emittance is dwindling because of the rapid power decline under its *T*^4^ dependence. Therefore, the hemispherical emittance at cryogenic temperatures is not likely to limit the thermal control performance. It also proves that focusing on the solar reflectance within the broadband region is quite reasonable for the cryogenic thermal control coatings.

Under the irradiance of the AM0 solar simulator, the bi-layer PRM meta-composite exhibits exceptional thermal control capability for cryogenic regulation. We set the sample in the home-built space simulator and monitored its temperature variance (Fig. 4c). Through the heat exchange, the sample reached the steady state with the equilibrium temperature as low as 123 K. Then, the solar simulator was turned on and beamed onto the suspended sample through the quartz window. The temperature of the bi-layer PRM meta-composite continued to rise under the heating of the simulated solar radiation, and finally stabilized at 145 K. We also took the alumina disks with a thickness of 500 μm for comparison, and the equilibrium temperature was as high as 205 K (Fig. S15b and c). The bi-layer PRM meta-composite demonstrates a remarkable 60 K improvement over the alumina reference sample due to the rejection of the input energy via high reflectance across the broadband solar irradiance spectrum. To evaluate the influence of the experimental errors, the theoretical thermal control performance of the bi-layer PRM meta-composite was calculated based on its experimental spectrum (Fig. 4d). When the cooling power is zero, the bi-layer PRM meta-composite reaches the steady state, and the theoretical equilibrium temperature is 152 K, being close to the experimental results. Compared to the reported thermal control coatings based on their measured optical properties^14,19,45–47^, the equilibrium temperature of our bi-layer PRM meta-composite is the lowest (Fig. 4e). It is worth noting that most coatings, aiming at reflecting solar irradiance within 0.2–2.5 μm, exhibit relatively high equilibrium temperatures exceeding 200 K with solar absorptance (*α*_s_, see details in the “Materials and methods” section) above 0.1. These results highlight the great potential of the bi-layer PRM meta-composite for cryogenic thermal control applications through innovative ultra-broadband reflectance (within the region of 0.2–8 μm), particularly compared to state-of-the-art coatings.

### Ground-based space irradiation tests and properties degradation

**Fig. 5:**
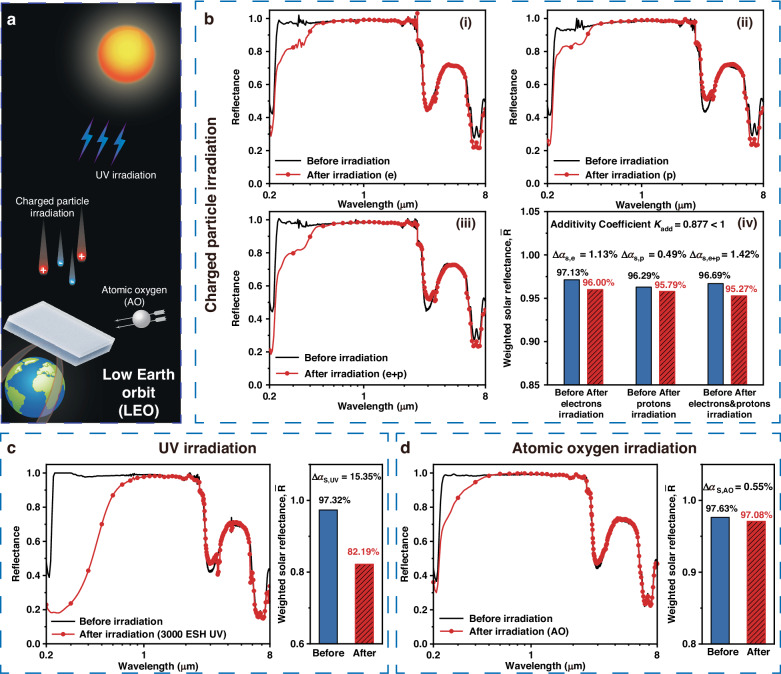
Degradation of optical properties of the bi-layer meta-composite after ground-based space irradiation tests. **a** Typical space environmental threats in LEO; **b** Optical degradation of bi-layer PRM meta-composite after charged particles irradiation, including reflectance spectra comparisons between pristine samples and ones after electrons irradiation (i), protons irradiation (ii), electrons & protons irradiation (iii) and variations in $\bar{R}$ (iv); **c** Reflectance spectra comparison and variation in $\bar{R}$ of the sample in UV irradiation test; **d** Reflectance spectra comparison and variation in $\bar{R}$ of the sample in AO irradiation test

The bi-layer PRM meta-composite exhibits outstanding thermal control performance under the AM0 solar irradiance, as we demonstrated. Following the mounting method of Optical Solar Reflectors (OSR)^48^, the meta-composite can be attached to exterior surfaces of spacecraft by silicone resins or other adhesives, enabling the space cryogenic thermal control. However, the impact of space environment always leads to optical properties degradation, thereby affecting thermal control performance^49^. On-orbit spacecraft are subjected to many environmental threats, including atomic oxygen (AO), charged particle irradiation, UV irradiation, solar irradiance, cosmic rays, and more^49,50^. Although all of these factors can cause the degradation of thermal control coatings, charged particles, AO, and UV irradiation are commonly regarded as the main threats in low Earth orbit (LEO)^51^ (Fig. 5a). To evaluate the durability and stability of our bi-layer meta-composite systematically, the ground-based simulated charged particles, UV, and AO irradiation tests have been conducted to characterize the properties degradation.

The charged particle irradiation, typically electrons and protons flux, is a harsh threat to the durability of thermal control coatings in the space environment^7^. After incident electrons strike the coatings, they could produce the ionizing effects when the kinetic energy of the collided electrons exceeds the binding energy of atomic nuclei^52^. And injected protons, through direct Coulomb interactions with atomic nuclei, cause displacement damage effects by dislocating lattice atoms from their normal equilibrium positions^53,54^. To evaluate the radiation resistance of our bi-layer PRM meta-composite, we performed systematic charged particle irradiation experiments with direct exposure to the electron and proton fluxes. The samples were tested under three different experimental conditions: (1) electrons irradiation only (e); (2) protons irradiation only (p); (3) combined electrons and protons irradiation (e + p). Irradiation tests used 50 keV electrons (fluence: 10^14^ e cm^-2^) and 50 keV protons (fluence: 10^12^ p cm^-2^) conditions simulating about 4 years of exposure in low Earth orbit^55^. The details are provided in the “Materials and methods” section.

The reflectance spectra were measured before and after charged particle irradiation for comparison (see Fig. 5b). All the degradation in reflectance of the bi-layer PRM meta-composite occurs in the UV region (<0.4 μm). The reflectance spectra in the region of 0.4–2.5 μm, where the solar irradiance is concentrated, were almost unaffected after these tests. This selective degradation in the UV region stems from irradiation-induced defect formation. The ionizing effects and displacement effects produced by injected charged particles were able to generate the oxygen vacancies, which are known as color centers^9^. And additional energy levels were introduced into the electronic structure during the irradiation, leading to the absorption edges shifting to longer wavelengths^56^. Although the UV region accounts for only about 8% of total solar irradiance, this portion still represents substantial energy. We therefore quantified the optical degradation through solar absorptance variation (Δ*α*_s_) and the additivity coefficient (*K*_add_) calculated by the Eqs. (1) and (2) to further evaluate the resistance^50^

1 $$\Delta {\alpha }_{{\rm{s}}}=(1-{\bar{R}}_{\mathrm{EOT}})-(1-{\bar{R}}_{\mathrm{BOT}})={\bar{R}}_{\mathrm{BOT}}-{\bar{R}}_{\mathrm{EOT}}$$

2 $${K}_{\mathrm{add}}=\frac{\Delta {\alpha }_{{\rm{s}},({\rm{e}}+{\rm{p}})}}{\Delta {\alpha }_{{\rm{s}},{\rm{e}}}+\Delta {\alpha }_{{\rm{s}},{\rm{p}}}}$$

where ${\bar{R}}_{{\rm{E}}{\rm{OT}}}$ and ${\bar{R}}_{{\rm{B}}{\rm{OT}}}$ represent the weighted solar reflectance of samples at the end and beginning of the tests. The parameter Δ*α*_s_ can also be seen as the variation in weighted solar reflectance.

As shown in Fig. 5b(iv), all samples exhibit less than 1.5% solar absorptance increase after charged particle irradiation. Notably, these results suggest that the bi-layer PRM meta-composite is still capable of reflecting most of the solar irradiance and maintains excellent thermal control performance despite exposure to the harsh particle bombardment. The additivity coefficient further provides critical insight into the irradiation resistance under combined electron and proton fluxes. When *K*_add_ = 1, the optical properties degradation represents simple additive damage from separate electron and proton fluxes, indicating no synergetic effects between incident charged particles. And *K*_add_ > 1 signifies the enhanced degradation beyond the summation of individual flux damage, arising from charged particles accumulation beyond ionizing effects and displacement effects. Synergetic effects between the injected charged particles induce more defects in the normal lattice. Most significantly, the observed additivity coefficient *K*_add_ < 1 in Fig. 5(b) demonstrates the recovery mechanism that prevents irradiation-induced optical degradation. This recovery, produced by synergetic effects, likely originates from the charge neutralization and defect recombination processes. This behavior not only confirms the exceptional irradiation tolerance of the bi-layer PRM meta-composite but also establishes its reliability for long-term space missions where combined charged particle irradiation is inevitable.

A comparison of the reflectance spectra before and after UV irradiation is displayed in Fig. 5c. UV irradiation was performed over a wavelength range of 0.2–0.4 μm for a total duration of 3000 equivalent sun hours (ESH) (see more details in the “Materials and methods” section). Since the photon energy in the UV irradiation test can reach up to 6.2 eV (corresponding to a wavelength of 0.2 μm), high-energy photons can directly excite electrons in the material. This induces severe ionizing effects and generates lattice point defects^57^. These defects could accumulate and act as color centers, absorbing solar irradiance in the UV and even visible region, which is the primary reason for the optical degradation observed in the bi-layer meta-composite. The comparison of the reflectance spectra demonstrates that UV irradiation causes more serious damage to the bi-layer meta-composite than charged particles. The spectral reflectance of the irradiated sample begins to decrease from around 1 μm, and the $\bar{R}$ across 0.2–8 μm reduces by 15.13%. However, due to the high${\,\bar{R}}_{{\rm{BOT}}}$ (97.32%), after 3000 ESH of UV irradiation, the weighted reflectance remains at 82.19%. It is still comparable to some existing thermal control coatings, such as Z93-C55 (about 83%) and AZ2100 (about 82%)^58^. Furthermore, with prolonged UV exposure time, the weighted reflectance of the bi-layer meta-composite stabilizes with no significant further degradation (Figs. S16 and S17). Figure S18 shows weighted solar reflectance as a function of exposure time for the bi-layer meta-composite and some existing thermal control coatings based on particulate medium structures. The bi-layer meta-composite consistently maintains a higher $\bar{R}$ than every other coating throughout the UV irradiation period, an indicator of its superior durability of space thermal control performance for long-term missions.

The bi-layer PRM meta-composite composed of all-inorganic materials has high resistance to AO, which has been verified by the AO exposure with a total fluence of 1 × 10^20^ atoms cm^-2^ (see more details in the “Materials and methods” section). The reflectance spectra before and after irradiation demonstrate that the damage induced by AO to the bi-layer meta-composite is minimal (Fig. 5d), with only a 0.55% reduction in $\bar{R}$ across 0.2–8 μm. And the structural stability also demonstrates the high AO resistance of the bi-layer meta-composite, which can be quantified by the mass loss (Δ*m*_ratio_) and erosion yield (*E*_y_)^59^. The definitions of Δ*m*_ratio_ and *E*_y_ are included in SI. It can be seen that the AO caused very slight damage to the sample, with negligible mass loss after exposure (only 0.03196%). And the advantage of inorganic materials is also evidenced by the *E*_y_ (only about 1.2641 × 10^-25 ^cm^3^ atom^-1^), being significantly lower than that of polymer materials commonly used for space thermal control, such as Kapton (*E*_y_ about 3 × 10^-24 ^cm^3^ atom^-1^), Nylon 66 (*E*_y_ about 1.8 × 10^-24 ^cm^3^ atom^-1^), and some others^59^.

The emittance spectra before and after charged particles, UV, and AO irradiation are shown in Figs. S19–S21. These typical space environmental threats have almost no effect on the emittance properties of the bi-layer PRM meta-composite, evidenced by almost no degradation observed in spectral emittance. The increase in lattice defects induced by both charged particles and UV irradiation even results in a slight improvement in weighted emittance. Based on the experimental reflectance and emittance spectra, the cooling power and the equilibrium temperature of the bi-layer meta-composite after irradiation tests can be calculated (Fig. S22). The results confirm that our bi-layer meta-composite still retains its space thermal control capability, despite the degradation of optical properties due to irradiation.

In addition to evaluating space irradiation resistance, the practical deployment schemes of the bi-layer meta-composite were also investigated. Considering that the bi-layer PRM meta-composite is a ceramic tile, it can be attached to the outer surfaces of spacecraft following the mounting method for OSR, as illustrated in Fig. S23. To verify the mechanical resilience of this ceramic coating, thermal cycling tests were conducted (see more details in SI). After 20 cycles of thermal cycling between −100 °C and 100 °C, no significant defects, such as spalling and delamination, were observed in the bi-layer meta-composite (Fig. S24a). And both experimental reflectance and emittance spectra remained consistent with those of the pristine sample before the test (Fig. S24b and c).

To meet the demand of low solar absorptance, we designed the bi-layer PRM meta-composite based on the scattering regime matching to realize ultra-broadband reflectance within 0.2–8 μm, instead of conventional 0.2–2.5 μm. This innovative spectrally selective response inspires a new strategy for space cryogenic thermal control. In the simulated space environments, the bi-layer PRM meta-composite obtained by the tape-casting process exhibits unprecedented cryogenic thermal control performance and resistance to charged particles, UV, and AO irradiation, which suggests the practicality in space applications.

## Discussion

Addressing the critical demand for spacecraft cryogenic thermal management, we have designed and developed a hierarchical PRM meta-composite, which achieves 97.3% weighted solar reflectance within the extended spectral region of 0.2–8 μm through the innovative scattering regime matching principle. By vertically integrating two PRM layers with precisely controlled scatterer sizes (*r*_eff_ = 0.7, *v*_eff_ = 0.3 in the upper layer and *r*_eff_ = 8, *v*_eff_ = 0.3 in the lower layer), the meta-composite achieves the spectrally selective complementary scattering and maximizes reflectance for the solar spectrum. Experimental validation demonstrates the remarkable thermal regulation capability under simulated space conditions, reaching an equilibrium temperature of 145 K under AM0 solar irradiance (1366 W m^-2^). This hierarchical PRM meta-composite exhibits a significant advancement in space cooling performance over existing passive thermal control coatings. The all-inorganic composition further ensures outstanding durability against charged particles and atomic oxygen irradiation, as evidenced by an optical degradation of less than 1.5% following ground tests. And even after long-term UV irradiation, the bi-layer meta-composite still maintains optical properties comparable to existing thermal control coatings. This irradiation resistance, combined with the inherent scalability of the tape-casting fabrication method, positions the bi-layer meta-composite as a practical solution for large-scale spacecraft applications. By modifying materials and structures, enhancing UV resistance, and validating the performance upon integration with spacecraft hardware, we will advance the suitability of the bi-layer PRM meta-composite for long-duration space missions. Beyond its thermal control applications, the demonstrated hierarchical design principle and scattering regime matching approach developed here offer universal strategies for creating broadband spectral responses and may find broader utility in diverse fields.

## Materials and methods

### Materials

The raw materials involved in preparation are all commercial and used without further processing. Yttrium oxide (Y_2_O_3_, >98.5%) particles with different sizes were purchased from the Beijing Zhongke Keyou Technology Co., Ltd. The co-polymers of isobutylene and maleic anhydride (Isobam-104 with a molecular weight of 55,000–65,000) were the commercial dispersant and purchased from the Kuraray Co., Ltd., Japan. The polyvinyl alcohol (PVA 1788) and n-butanol (>99.5%, GC) were both purchased from the Shanghai Aladdin Co., Ltd., and some other solvents and additives (such as ethanol (AR)) were purchased from Shanghai Titan Technology Co., Ltd.

### Bi-layer PRM fabrication

The bi-layer PRM meta-composite here was fabricated via the dual tape-casting and one-step sintering. Firstly, the large-scale Y_2_O_3_ particles were mixed with deionized water containing an appropriate amount of dispersant to obtain the particle content close to 78.8 wt%. The dispersant was Isobam-104 and 0.2 wt% regarding the particle content. Then the binder (polyvinyl alcohol, PVA) and defoamer (n-butanol) were added to the slurry. The slurry was stirred thoroughly by the centrifugal to ensure the particles were homogeneous and stable in suspension. The slurry of the small-scale Y_2_O_3_ particles was also prepared following the above steps, with the particle content close to 75 wt% and 0.3 wt% dispersant regarding the particles. The binder and defoamer were also PVA and n-butanol here.

The prepared slurry of the large-scale particles was cast on the flat tape, and the thickness can be controlled by the gap between the blade and the polymer tape. After drying at room temperature for 12 h, the slurry of small-scale particles was poured onto the mono-layer PRM and cast by the blade immediately. The thickness of this upper layer was also controlled by the gap. After 12 h drying, the sintering process was conducted from room temperature to 600 °C at a rate of 5 °C min^-1^ and held for 2 h to burn off the temporary organic dispersant and binder completely. Then, the temperature was raised to 1200 °C and held for 6 h to ensure the sintering necks formed between Y_2_O_3_ particles.

### Structure and optical characterization

To observe the morphology, the samples were set into the scanning electron microscope (Gemini 300, Zeiss). The particle sizes of small-scale particles were measured by the Mastersizer 2000E, Malvern. The pore size distributions and the corresponding volume fraction were measured via mercury intrusion porosimetry (MIP) using a Micromeritics Autopore v9600 Mercury Porosimeter. Compressive strength measurements were performed on cubic samples (abraded into 10 mm × 10 mm× 10 mm with a dimensional tolerance within ±0.2 mm) after sintering, at a crosshead speed of 0.5 mm min^-1^ (Instron 5967).

The reflectance of samples within 0.2–2.5 μm was tested using an ultraviolet/visible/near-infrared spectrometer (Lambda 950, Perkin Elmer). And the reflectance within 2.5–8 μm was characterized using a Fourier Transform Infrared (FTIR) Spectroscopy (Nicolet 6700, Thermo Fisher Scientific). Based on the experimental spectral reflectance, the weighted solar reflectance from 0.2 μm to 8 μm can be calculated by the equation $\bar{R}=\frac{{\int }_{0.2}^{8}R(\lambda ){I}_{{\rm{AM}}0}(\lambda )d\lambda }{{\int }_{0.2}^{8}{I}_{{\rm{AM}}0}(\lambda )d\lambda }$, where *R*(*λ*) is the spectral reflectance corresponding to wavelength *λ* and *I*_AM0_(*λ*) is the standard solar spectral irradiance^6^. Because the samples all had no transmittance, the spectral emittance from 8 μm to 16 μm can be calculated by emittance = 1 − reflectance after being characterized by the FTIR Spectroscopy with the integrating sphere. And the weighted mid-infrared emittance can be defined as $\bar{\varepsilon }=\frac{{\int }_{8}^{16}\varepsilon (\lambda ,T){I}_{{\rm{BB}}}(\lambda ,T)d\lambda }{{\int }_{8}^{16}{I}_{{\rm{BB}}}(\lambda ,T)d\lambda }$, where *ε*(*λ, T*) is the spectral emittance corresponding to *λ* and temperature *T*, *I*_BB_(*λ, T*) is the ideal blackbody radiation^6^. The X-ray photoelectron spectroscopy (XPS) results were obtained by the Thermo Scientific K-Alpha XPS System. The thermal control performance was characterized by a home-built dual-function space simulator.

### Ground-based space irradiation tests

The charged particle irradiation tests were performed on the Space Irradiation Simulation Equipment in the Shanghai Institute of Spacecraft Equipment. Taking low Earth orbit as the target, the energy of charged particles was set as 50 keV because the electron and proton spectrum inside the radiation belt of the Earth possessed the maximum value of the flux corresponding to tens of keV^60^. Considering the resistance of thermal control coatings to charged particles is actually assessed by the received irradiation fluences. The total fluence of electrons and protons was selected to simulate the environmental effects of low Earth orbit for about 4 years^55^. The samples were tested with three different conditions: (1) electrons irradiation of 1 × 10^14^ e cm^-2^ fluence with an energy of 50 keV; (2) protons irradiation of 1 × 10^12^ p cm^-2^ fluence with an energy of 50 keV; (3) electrons and protons comprehensive irradiation of 1 × 10^14^ e cm^-2^ fluence with an energy of 50 keV for electrons and 1 × 10^12^ p cm^-2^ fluence with an energy of 50 keV for protons. And the flux of electrons and protons was set as 5 × 10^10^ e (cm^2^ × s)^-1^ and 5 × 10^9^ p (cm^2^ × s)^-1^ in this work.

UV irradiation tests were also performed on the Space Irradiation Simulation Equipment in the Shanghai Institute of Spacecraft Equipment. The UV irradiation test covered a wavelength range of 0.2–0.4 μm. The exposure was accelerated by selecting equivalent UV sun intensity greater than one sun, and monitored with time up to 3000 ESH. UV irradiation tests were carried out in a vacuum of 1 × 10^-4 ^Pa.

AO irradiation tests were conducted on the SimulTek Compact AO system (SimulTek Research Co., Ltd.). The average energy of the AO was set to about 5 eV, similar to the real AO energy in LEO. The AO exposure was in the vacuum of 2.5 × 10^-2^ Pa with a flux of 5 × 10^15^ atoms (cm^2^× s)^-1^. And a total AO fluence of 1 × 10^20^ atoms cm^-2^ was obtained by keeping the exposure time up to about 6 h.

### Equilibrium temperature and cooling power calculation

The temperature of the thermal control coatings while the spacecraft at the thermal equilibrium state is the equilibrium temperature in the real on-orbit situation. To simplify the thermal analysis, there is a universal assumption that only solar irradiance and thermal radiation are taken into account. As for a surface with spectrally selective reflectance in the space, the equilibrium temperature is determined by a balance between heat lost through thermal radiation (*Q*_R_) and heat absorbed from the solar irradiance (*Q*_A_). Without the thermal conduction, the equilibrium of energy is expressed as *Q*_A_ = *Q*_R_. The power of thermal radiation and the absorptance from the solar irradiance can be calculated by^45^

3 $${Q}_{{\rm{A}}}={\alpha }_{{\rm{s}}}\times S\times {A}_{{\rm{S}}}$$

4 $${Q}_{{\rm{R}}}=\varepsilon \times \sigma \times {T}^{4}\times {A}_{{\rm{R}}}$$

where *A*_S_ is the area of surface exposed to the incident solar irradiance, *σ* the Stefan-Boltzmann constant (5.67 × 10^-8 ^W m^-2^ K^-4^), and *A*_R_ the area of surface for thermal radiation exposed to the space. Here, *α*_s_ is the solar absorptance, which can be calculated by^45^

5 $${\alpha }_{{\rm{s}}}=\frac{{\int }_{{\lambda }_{1}}^{{\lambda }_{2}}(1-R(\lambda )){I}_{{\rm{AM0}}}(\lambda )d\lambda }{{\int }_{{\lambda }_{1}}^{{\lambda }_{2}}{I}_{{\rm{AM0}}}(\lambda )d\lambda }$$

where *λ*_1_ and *λ*_2_ are the smallest and largest wavelength of the considered reflectance spectrum region. It is noteworthy that the *α*_s_ of non-transparent coatings such as our bi-layer meta-composite can be calculated by the relationship that ${\alpha }_{{\rm{s}}}=1-\bar{R}$.

Therefore, the equilibrium temperature of the surface is shown in^45^

6 $${T}_{{\rm{e}}}=\root{{4}}\of{\frac{{\alpha }_{{\rm{s}}}\times S\times {A}_{{\rm{S}}}}{\varepsilon \times \sigma \times {A}_{{\rm{R}}}}}$$

It is evident from Eq. (6) that the equilibrium temperature (*T*_e_) is only related to the solar absorptance and emittance of thermal control coatings. Thus, *T*_e_ is considered as one effective quantity to characterize the thermal control performance of different coatings.

The cooling power of the surface (*P*_cooling_) is expressed simply as^17^

7 $${P}_{{\rm{cooling}}}={P}_{{\rm{rad}}}(T)-{P}_{{\rm{solar}}}$$

*P*_rad_ the radiation power and *P*_solar_ solar absorption power can be defined as^17^

8 $${P}_{{\rm{rad}}}(T)={A}_{{\rm{R}}}\int d\varOmega \,\cos \,\theta {\int }_{0}^{\infty }d\lambda {I}_{{\rm{BB}}}(T,\lambda )\varepsilon (\lambda )$$

9 $${P}_{{\rm{solar}}}={A}_{{\rm{S}}}{\int }_{0}^{\infty }d\lambda \varepsilon (\lambda ){I}_{{\rm{AM0}}}(\lambda )$$

When *P*_cooling_ = 0, there is no heat transfer flux input or output to the surface, and the equilibrium temperature is reached, which is almost the same as the calculated result of Eq. (6).

## Supplementary information

Supplementary Information

## Data Availability

The data in this study are available from the corresponding author upon reasonable request.
