# Supplementary material for: Bi-layer photonic random meta-composite for cryogenic thermal control by ultra-broadband scattering matched reflectance
Source: Light Sci Appl. 2026 Jun 30;15:298. doi: 10.1038/s41377-026-02372-9 (PMC13319754; doi:10.1038/s41377-026-02372-9)
Supplement: Supplementary file 1 — Supplementary Information [file 41377_2026_2372_MOESM1_ESM.docx]

***Supplementary Information for***

**Bi-layer photonic random meta-composite for cryogenic thermal control by ultra-broadband scattering matched reflectance**

Hongchao Li^1^, Hexiang Han^2^, Zhiyuan Zhao^1^, Hao Gong^1^, Xiaokun Song^1^, Zhongyang Wang^1, *^, Gang Liu^2^, Tongxiang Fan^1, *^, Xiao Zhou^1, *^ and Di Zhang^1^

*^1^ State Key Laboratory of Metal Matrix Composites, School of Materials Science and Engineering, Shanghai Jiao Tong University, Shanghai 200240, China*

*^2^ Shanghai Institute of Spacecraft Equipment, Shanghai 200240, China*

**Section S1: Particulate medium simulations**

**The double optimized Monte Carlo simulation**

The double optimized Monte Carlo (MC) method was proposed by us in the previous article^1^. The pristine MC method is performed on the assumptions of independent scattering and mono-disperse conditions. To effectively predict optical properties of dense and poly-disperse particulate media, we have already developed a framework to refine the MC method by introducing size distributions and dependent scattering. Particle distributions can be featured by two structural parameters: the effective radius (*r*_eff_) and the effective variance (*v*_eff_), which are expressed as^2^

$r_{\text{eff}}=\frac{1}{G}\int_{r_{1}}^{r_{n}} r\pi r^{2}n(r)dr$ (S1)

$v_{\text{eff}}=\frac{1}{Gr_{\text{eff}}^{2}}\int_{r_{1}}^{r_{n}} \left(r-r_{\text{eff}}\right)^{2}\pi r^{2}n(r)dr$ (S2)

$G=\int_{r_{1}}^{r_{n}} \pi r^{2}n\left(r\right)dr$ (S3)

*n*(*r*)d*r* is the probability of particles with radius between *r* and *r*+d*r*. *r_1_* and *r_n_* are the smallest and largest particles in the size distribution. *G* represents the mean geometric cross-sectional area of particles. In this work, the *r*_eff_ and *v*_eff_ of particles in upper and lower particulate media layers were calculated by the Equation (S1) and (S2). The probability density functions, *n*(*r*), were the experimental results as shown in **Fig. S3**. Then, the polydisperse particulate media were simplified to the mono-sized system and the dependent scattering factors could be calculated on the basis of *r*_eff_ and *v*_eff_. The computational method was described in details^1^.

The MC simulations fed by double optimized scattering properties were further utilized for calculating reflectance of layers composed of small-scale particles in **Fig. 3(a)**, **(b)** and **(c)** in the manuscript. The particles volume fraction was set as 55%. The thickness varied. The refractive index of particles was set as that of Y_2_O_3_ and the refractive index of surrounding medium, namely air, was fixed as 1. At the beginning of the MC simulations, a large number of photons were set above the particulate medium initially and emitted into the particulate medium normally. When the photons propagated between particles, the trajectory can be recorded. Because the weight of every photon was initialized to 1, the optical properties such as reflectance, transmittance can be obtained by counting the weights and positions of photons exiting the particulate medium. We launched 500000 photons in every simulation.

**The Geometric Optics Monte Carlo simulation**

The *r*_eff_ and *v*_eff_ of large-scale particles in lower particulate media were 8.59 and 0.29 calculated by Equation (S1), (S2). The corresponding size parameter is so large that dependent scattering correction proposed by Tien and co-workers is invalid in MC method^3,4^. Considering that geometric scattering regime dominates in the most region from 0.2 μm to 8 μm according to the scattering regime matching principle, the MC simulation based on the ray tracing model is suitable to obtain the optical properties of the large-scale particles media^5,6^. As displayed in **Fig. S11(a)**, the scattering of photons by a transparent or semi-transparent particles is described in terms of multiple reflections and transmissions. The reflectance and transmittance at interfaces between particles and surrounding media can be calculated by the Fresnel laws. And the random number decides the behavior of the photon when it strikes a particle. If the random number is less than the reflectance, the light is reflected at the exterior or interior interface of particles. Otherwise, the transmission occurs. Generally, tracing up to three times interactions is sufficient.

We carried out the geometric optics MC simulations for spectra in **Fig. 3(c)** in the manuscript as mentioned above. The particulate medium composed of large-scale Y_2_O_3_ particles was established first by the sequential addition method with volume fraction *f*_v_ = 50%^7^. The surrounding medium was also the air. Particle distribution was kept the same as the experimental results in **Fig. S3**. The size in x-y plane of the particulate medium model was 81 μm × 81 μm. The thickness along z axis was set as 500 μm. The periodic boundary condition was adopted at the sides of x-y plane. The schematic of the calculation model established for geometric optics MC simulations is shown as **Fig. S11(b)**. We launched 50000 photons above the particulate medium initially and emitted into the particulate medium normally. Each photon transferred in a straight line. When the photon was intercepted by a particle, three interactions were considered and the position where the photon exited the particle can be determined. Then, the photon kept propagating in air and hit other particles. Tracing each photon until it left the particulate medium or its weight reduced to 0. It was worth noting that the weight of photon was reduced because of the particle absorption in the case of semi-transparent particles, which can be calculated by the Beer-Lambert law. **Figure S12** is the flowchart of the geometric optics MC simulation.

To validate the geometric optics MC simulation, a mono-layer particulate medium consisting of large-scale particles was prepared experimentally, with 50% volume fraction and 500 μm thickness. **Figure S11(c)** compared its experimental spectrum with the reflectance spectrum calculated by the geometric optics MC method. It suggested the validation of this modified MC method for particulate media where geometric regime dominated.

**The continuum model combining the two types of Monte Carlo simulations**

The reflectance spectrum of bi-layer PRM meta-composite in **Fig. 3(c)** was calculated based on a continuum model. This continuum model combined the double optimized MC and geometric optics MC simulation. The flowchart of this continuum calculation model is shown as **Fig. S13**.

The photons with the unit weight were emitted normally into the upper PRM layer initially. The interactions between the photon and small-scale Y_2_O_3_ particles were determined by the double optimized scattering characteristics, taking dependent scattering and size distributions into account. The photons kept transferring in the upper layer until it reaches the boundary. When the photon hit the upper boundary of the current layer, it entered the air and the weight was scored into reflectance. When the photon hit the lower boundary, it entered the large-scale PRM layer and continued propagation with the current direction and weight until reaching the boundary. The weights of photons hitting the lower boundary of the large-scale PRM layer were registered as transmittance. And the photons hitting the upper boundary of the large-scale PRM layer transferred into the small-scale PRM layer again.

To accomplish this continuum transfer of photons, we combined the codes of the double optimized MC and geometric optics MC method. The propagation of photon in the upper or lower PRM layers was simulated by the corresponding code. When the photon passed the interface between two layers, the direction cosines and weight were taken as the input and the propagation was simulated by another code. The bi-layer PRM meta-composite in **Fig. 3(c)** in the manuscript was composed of a 50 μm thickness small-scale PRM layer and a 500 μm thickness large-scale PRM layer. The Y_2_O_3_ particle distributions in these two layers were *r*_eff_ = 0.7, *v*_eff_ = 0.3 and *r*_eff_ = 8, *v*_eff_ = 0.3, respectively. The surrounding medium was air. And the particle volume fractions of the upper layer and the lower layer were set as 55% and 50%. We also launched 50000 photons and emitted them into the bi-layer meta-composite normally. The reflectance spectrum calculated by the continuum MC method was the red line with diamond marks in **Fig. 3(c)**.

**Light scattering analysis**

The scattering efficiency and phase function were calculated based on the Mie theory^8^. We assumed the refractive index of the non-absorbing particles as 2+0i for simplification. And the surrounding medium was air.

The scattering efficiency of a single particle was expressed as^8^

$Q_{\text{sca,single}}=\frac{2}{x^{2}}\sum_{n=1}^{\infty} (2n+1)\left(a_{n}a_{n}^{*}+b_{n}b_{n}^{*}\right)$ (S4)

where *x* was the size parameter, complex numbers *a_n_* and *b_n_* the Mie coefficients related to the refractive index and radius of the particle^8^. The superscript * represented the conjugate of complex numbers. Taking the size distributions of small-scale and large-scale particles into account, we calculated the scattering efficiency of the particle clouds further. And the expression was^2^

$Q_{\text{sca}}=\frac{\int_{r_{1}}^{r_{2}} \pi r^{2}Q_{\text{sca,single}}(r)n(r)\text{d}r}{G}=\frac{\int_{r_{1}}^{r_{2}} \pi r^{2}Q_{\text{sca,single}}(r)n(r)\text{d}r}{\int_{r_{1}}^{r_{2}} \pi r^{2}n(r)\text{d}r}$ (S5)

where *Q*_sca_ was the scattering efficiency of a particle could whose size distribution was defined by *r*_eff_ and *v*_eff_.

The phase functions calculated by Mie theory in **Fig. 3(e)** and **(f)** were displayed in terms of polar graphs. They were capable of exhibiting the probability distributions of scattered fields clearly in the three-dimensional space when the plane wave with a certain wavelength was incident on a single particle. And the details of the phase functions calculation are referred to read the reference^8^.

**Section S2: Sintering Conditions and Compressive Mechanical Properties**

The bi-layer meta-composite proposed by us consists of two dense Y_2_O_3_ particulate media. Note that the small- and large-scale Y_2_O_3_ particles are not simply packed together. During the high-temperature sintering, the formation process of necks between particles are facilitated to create rigid bonding, which not only fixes particle positions in particulate media but also ensures that the bi-layer meta-composite obtains sufficient mechanical properties for mounting applications. The scanning electron microscope (SEM) and compressive tests were carried out to demonstrate the effective bonding between particle and illustrate the structure stability of the bi-layer meta-composite.

**Samples Preparations and Characterization Methods**

The slurry consisting of small-scale Y_2_O_3_ particles, whose *r*_eff_ and *v*_eff_ are 0.7 and 0.3, with the same composition as used in tape casting of bi-layer meta-composite was prepared by centrifugal dispersion. Then, the cubic green bodies with dimensions of no less than 10 mm × 10 mm × 10 mm were produced by drop casting using a plastic mold. The green bodies were sintered at 1050 ℃, 1200 ℃, and 1350 ℃ for 6 hours, respectively. After sintering, the samples were abraded into 10 mm cubes with a dimensional tolerance within ±0.2 mm. The compressive measurements of these sintered samples were performed by Instron 5967 at a crosshead speed of 0.5 mm min^-1^, while the strain along the compressive direction was measured according to the crosshead displacement. The maximum load reached during the test was used to calculate the compressive strength. And the micro-structures of each sample were characterized by the SEM (Gemini 300, Zeiss).

Compared to the small-scale Y_2_O_3_ particles, the large-scale particles with *r*_eff_ about 8 and *v*_eff_ about 0.3 in the lower layer exhibited lower sintering activity. Even a temperature of 1350 ℃ could not lead to the formation of sintering necks between the large-scale Y_2_O_3_ particles. Therefore, 5wt% of small-scale Y_2_O_3_ particles based on the total particle mass were added as a sintering aid in the preparation of the large-scale particle slurry. And the contents of organic additives were the same as the slurry for the lower layer tape-casting fabrication. The cubic green bodies prepared from this slurry via drop casting were sintered at 1200 ℃ for 6 hours, then abraded into 10 mm cubes with a dimensional tolerance within ±0.2 mm. The compressive strength and the micro-structure were also characterized as that of sintered samples composed of small-scale particles.

**Characterization Results and Analysis**

The SEM images and stress-strain curves of sintered samples composed of small-scale Y_2_O_3_ particles are exhibited in **Fig. S5**. With sintering temperature increasing, the necks size between particles grows obviously (**Fig. S5(a)**, **(b)** and **(c)**, scale bar: 2 μm), leading to stronger inter-particles connections and notable rise in compressive strength (**Fig. S5(d)**). It is noteworthy that the stress-strain curves were characterized by the plateau rather than the sudden rupture as generally recorded for dense ceramics. It can be attribute to the propagation of cracks in samples by necks fracture^9^. However, high-temperature sintering also results in more pronounced changes in geometries of particles. After 1350 ℃ sintering for 6 hours, some particles have fully coalesced, losing their light-scattering capability as an individual unit (**Fig. S5(c)**). Thus, 1200 ℃ was identified as the optimal sintering temperature for small-scale Y_2_O_3_ particles to balance the great light-scattering capability and sufficient neck size for mechanical properties and structural stability.

The SEM image and stress-strain curve of sintered samples composed of large-scale Y_2_O_3_ particles are exhibited in **Fig. S6**. After centrifugal dispersion, the large- and small-scale particles were uniformly mixed and the small-scale Y_2_O_3_ particles filled the gaps between large-scale particles. During sintering, the small-scale particles formed necks and connected adjacent large-scale particles (**Fig. S6(a)**, scale bar: 10 μm), thereby fixing their positions. The stress-strain curve of compressive test further confirmed the effective bonding realized by small-scale particles (**Fig. S6(b)**), although the strength was lower than that of the small-scale particle samples sintered at 1200 ℃ (**Fig. S5(d)**).

The ability of small-scale Y_2_O_3_ particles to act as a sintering aid and connect large-scale particles demonstrates that the upper and lower PRM in the bi-layer meta-composite are effectively bonded. As a result, the spatial distribution of particles within the bi-layer meta-composite is fixed rather than a simple stacking, ensuring structural stability and meeting the requirements for practical applications.

**Section S3: Vickers Microhardness (VHN) Analysis**

To verify the mechanical integrity of the bi-layer PRM meta-composite, Vickers microhardness (*VHN*) analysis was performed on 3 samples fabricated in different batches. These samples were all pre-cut in form of 40 mm × 40 mm and sintered at 1200 ℃ for 6 hours. Microhardness tests were carried out using a Micro Hardness Tester (XHVT-1000Z, Shanghai Shangcai Testing Machine Co., Ltd., China) with loads of 0.2 kg for 15 s. During testing, 5 positions were selected on each sample (**Fig. S7(a)**) and an average of 3 indents at each position was analyzed. The indentations diagonals were measured directly in the tester (**Fig. S7(b)**, scale bar: 200 µm) and the *VHN* can be obtained by^10^

$VHN=1854.4\frac{P}{d^{2}}$ (S6)

where *VHN* has the unit of kg mm^-2^, *P* is the applied load with the unit of gram and *d* is the indentation diagonal given in µm, which sometimes is the average of two indentation diagonal (*d_1_* and *d_2_*) when the indentation is not square.

The results of *VHN* tests are shown in **Fig. S7(c)**. Based on the indentation dimensions and geometry of the indenter, *VHN* tests could produce sufficient indentation depth to investigate the influence of the interface. Since the sintering temperature of the samples was controlled to preserve the geometric shape of particles, the bonding strength between particles was relatively weak, resulting in low *VHN* values in **Fig. S7(c)**. It can be indicated that, although the measured *VHN* fluctuates across different tests due to interfacial roughness and is not strictly uniform, the average *VHN* remains highly consistent across different samples and positions. This implies that while interfacial roughness affects the local mechanical properties of the bi-layer meta-composite, it still allows for overall mechanical integrity in large-scale fabrications and applications.

**Section S4: Thermal Control Performance Characterized by the space simulator**

The thermal control performance of bi-layer PRM was characterized by a dual-function space simulator (**Fig. 4(a)** in the main text). It is built for lab-scale experiments to characterize emittance and the thermal control performance by modifying the standard steady-state calorimetric equipment. The home-built space simulator creates the high vacuum environment (<10^-5^ Pa) and is cooled by liquid nitrogen continuously injected into the hollow vacuum chamber. The high emittance black paint that can maintain its optical properties at the cryogenic temperature are applied to the inner surface of the vacuum chamber to promote heat exchange between samples and the deep cryogenic background. The area ratio of the inner surface to the sample is greater than 100, in order to minimize the errors during the calorimetric process. A commercially available solar simulator acts as the irradiance source, creating broadband radiation as high as the AM0 solar constant (about 1366 W m^-2^) with a spectrum similar to that of the Sun (**Fig. S15(a)**). The spot of light beam is tuned as the same size as the sample suspended in the vacuum chamber. And the space simulator enables characterization of both the thermal control performance under the solar irradiance and temperature-dependent emittance via controlling the switch of the solar source.

Different from the spectral emittance and weighted emittance within a certain wavelength range, the hemispherical emittance exhibited in **Fig. 4(b)** is the value within the full spectrum and measured by the steady-state calorimetric method^11^. Keep the solar source off, the temperature of sample was maintained at the set value by a thin film heater. T-type thermocouples were placed on the front and back sides of the sample to monitor the temperature. Maintaining the background temperature at 77 K, the heating power of the heater was recorded. After the sample reaching thermal equilibrium, the output power of the heater was equal to the power radiated outward by the sample. And the hemispherical emittance of the sample is the ratio of the heating power to the ideal blackbody radiation power.

With the solar source turned on, the radiance is beamed onto the suspended sample through the quartz window. Under the irradiance of the AM0 solar simulator, the sample suspended in the space simulator is heated up. The temperature variance is monitored and displayed (**Fig. 4(c)** and **Fig. S15(c)**) to exhibit the thermal control performance in cryogenic space environment directly.

**Section S5: The Parameters for Evaluating Atomic Oxygen (AO) Resistance**

The AO in space environment degrades the thermal control materials via oxidation and particles collisions, resulting in the structure damage and mass variations^12^. To quantify the erosion of AO, there are two parameters used in this work, namely the mass loss (Δ*m*_ratio_) and erosion yield (*E*_y_) of the irradiated samples.

The Δ*m*_ratio_ is defined as

$\Delta m_{\text{ratio}}=\frac{\Delta M}{m_{\text{BOT}}}\times100\%=\frac{m_{\text{BOT}}-m_{\text{EOT}}}{m_{\text{BOT}}}\times100\%$ (S7)

where *m*_BOT_ the mass of the sample at the beginning of tests and *m*_EOT_ the mass at the end of tests. And *E*_y_, the volume of materials removed per incident oxygen atom, of the sample is determined by^13^

$E_{\text{y}}=\frac{\Delta M}{A_{\text{AO}}\rho_{\text{S}}F}$ (S8)

where *A*_AO_ the surface area of the sample exposed to AO, *ρ*_s_ the density of the sample and *F* the AO fluence. In our test, *A*_AO_ of our sample was 3 × 3 cm^2^, *ρ*_s_ of Y_2_O_3_ was about 5.01 g cm^-3^ and *F* was 1×10^20^ atoms cm^-2^. The Δ*m*_ratio_ and *E*_y_ of the irradiated sample in this work was calculated and recorded in **Table. S1** below.

Table. S1 Experimental results of AO exposure

| *m*_BOT_ (g) | *m*_EOT_ (g) | Δ*M* (g) | Δ*m*_ratio_ | *E*_y_ (cm^3^ atom^-1^) |
| --- | --- | --- | --- | --- |
| 1.78356 | 1.78299 | 0.00057 | 0.03196% | 1.2641×10^-25^ |

**Section S6: Supplementary Figures**


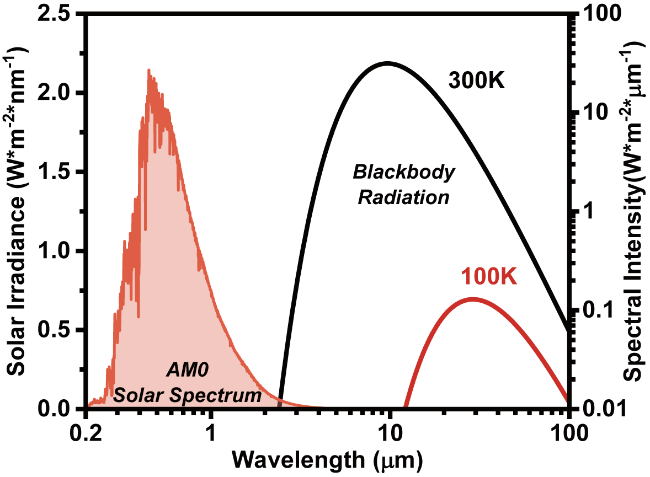


Fig. S1. The AM0 solar irradiance spectrum and the ideal blackbody radiation intensity at 100 K and 300 K.


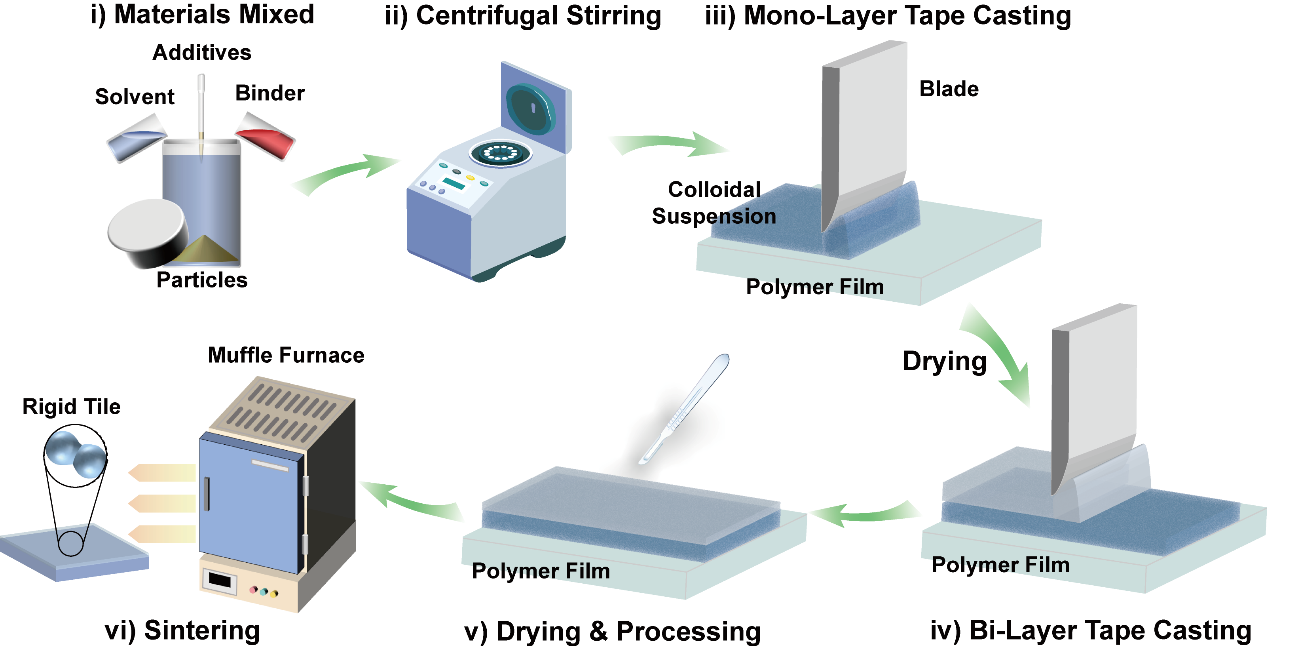


Fig. S2. Schematic of fabrication processes of the bi-layer PRM meta-composite.


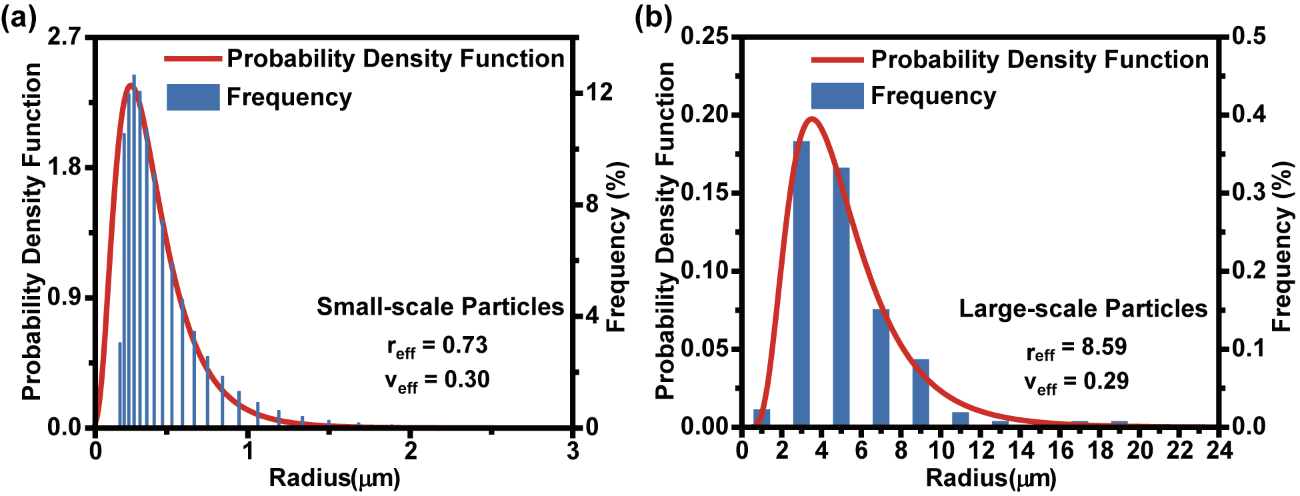


Fig. S3. (a) Size distribution of the small-scale Y_2_O_3_ particles in the upper layer (*r*_eff_ = 0.73 and *v*_eff_ = 0.3); (b) Size distribution of the large-scale Y_2_O_3_ particles in the lower layer (*r*_eff_ = 8.59 and *v*_eff_ = 0.29). Both are the log-normal distribution.

Table. S2. The experimental data of upper and lower PRM measured by the Archimedes method.

| Sample | Dry Weight of Sample in Air | Floating Weight of Sample in Water | Wet Weight of Sample in Air | Theoretical Density of Y_2_O_3_ | Volume Fraction, *f*_v_ |
| --- | --- | --- | --- | --- | --- |
| The Upper PRM | 1.4732 g | 1.0943 g | 1.6304 g | 5.01 g cm^-3^ | 54.85% |
| The Lower PRM | 2.0391 g | 1.6286 g | 2.4300 g |  | 50.79% |


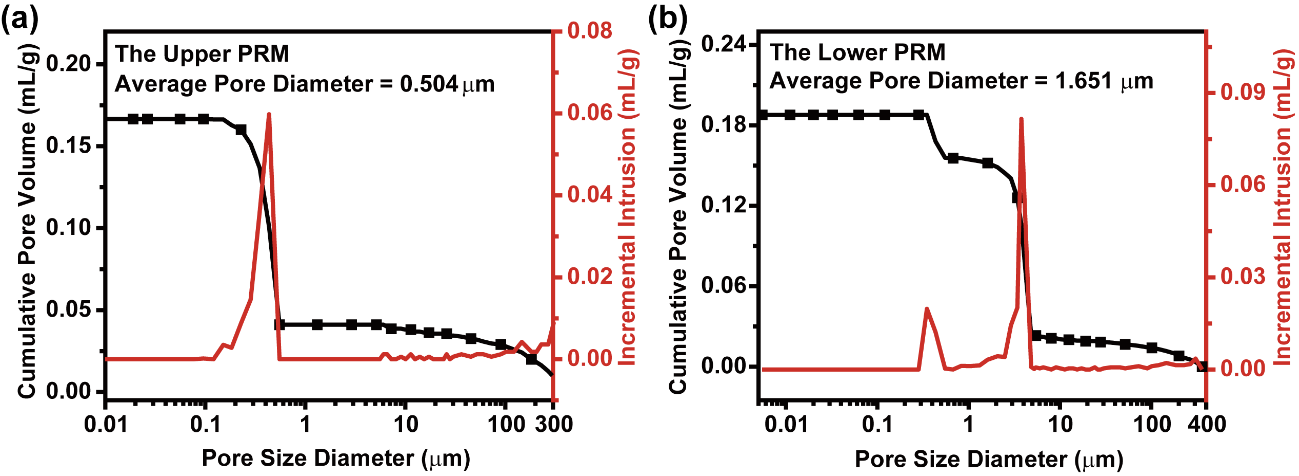


Fig. S4. Pore size distributions of the upper layer PRM composed of small-scale Y_2_O_3_ scatters (a) and the lower layer PRM composed of large-scale Y_2_O_3_ scatters (b).

To investigate the volume fractions of particles in upper and lower layers, we prepared the mono-layered PRM consisting of small-scale and large-scale particles respectively by the same recipes and methods as mentioned in the main text. The Archimedes method was carried out to test the volume fractions.^14^ The experimental data were listed in **Table. S2**. And we also evaluated the particles volume fractions by the mercury intrusion porosimetry (MIP). The relationships between intrusion volumes versus pore diameter size of the standalone upper and lower PRM are shown in **Fig. S4**. The average pore size of the upper layer PRM is 0.504 μm, which is much smaller than the 1.651 μm in the lower layer PRM. And the cumulative pore volume of the upper PRM is 0.1666 ml g^-1^ and that of the lower PRM is 0.1878 ml g^-1^. Thus, both Archimedes method and MIP method confirmed the volume fraction of the upper layer was about 55% and that of the lower layer was about 50%, which was as expected to keep the samples standalone without binders.


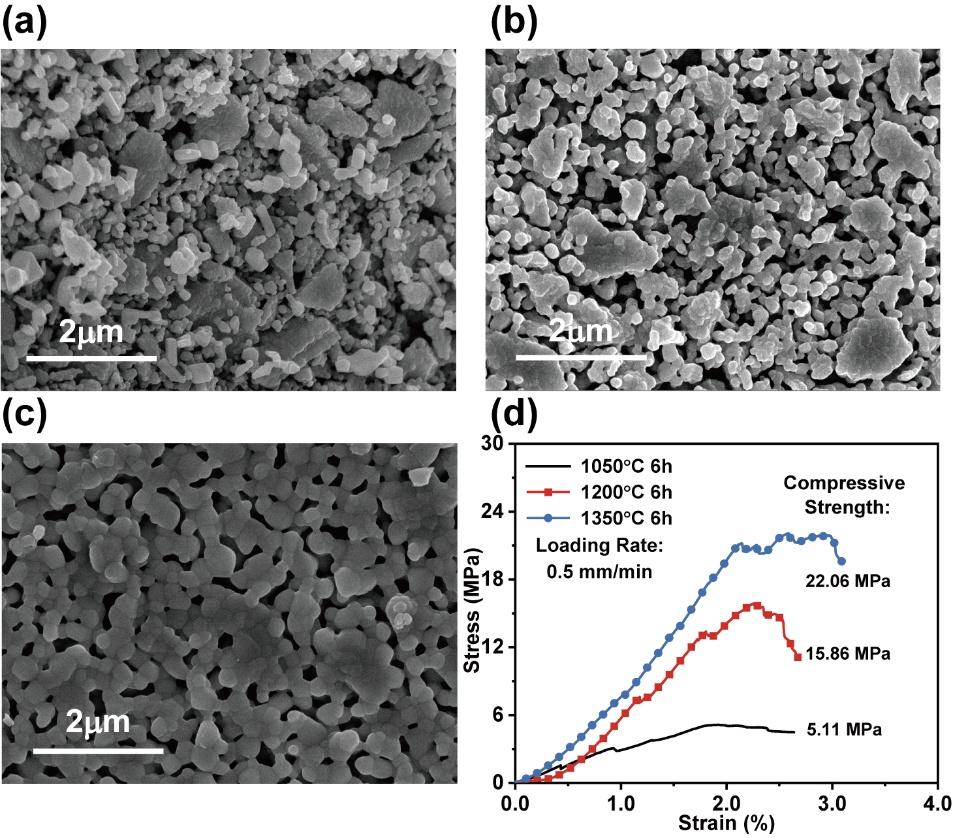


Fig. S5. The SEM images of cubic samples composed of Y_2_O_3_ small-scale particles sintered at (a) 1050 ℃, (b) 1200 ℃ and (c) 1350 ℃; (d) Stress-strain curves of cubic samples sintered at different temperature.


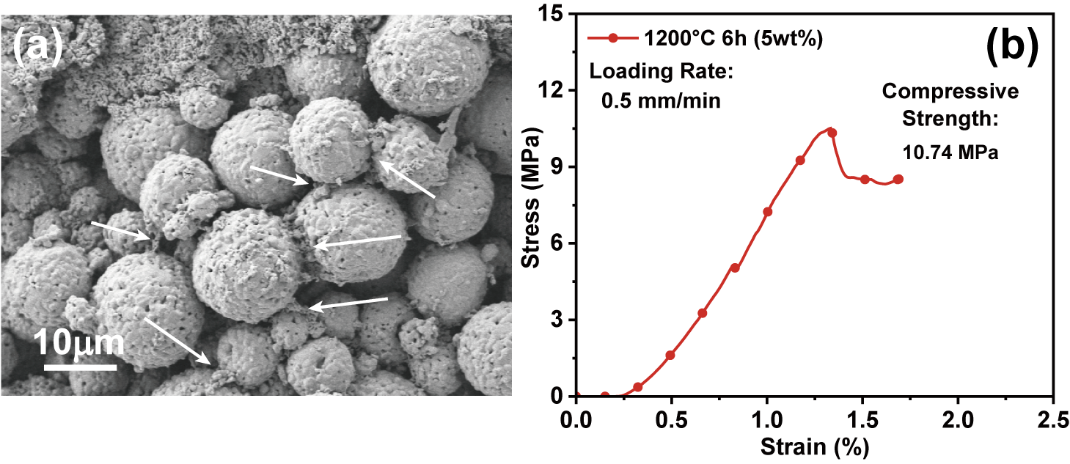


Fig. S6. (a) The SEM image and (b) stress-strain curve of the cubic sample composed of Y_2_O_3_ large-scale particles using small-scale particles as a sintering aid sintered at 1200 ℃ (Arrows: small-scale Y_2_O_3_ particles filled in gaps and made effective connections between large-scale particles).


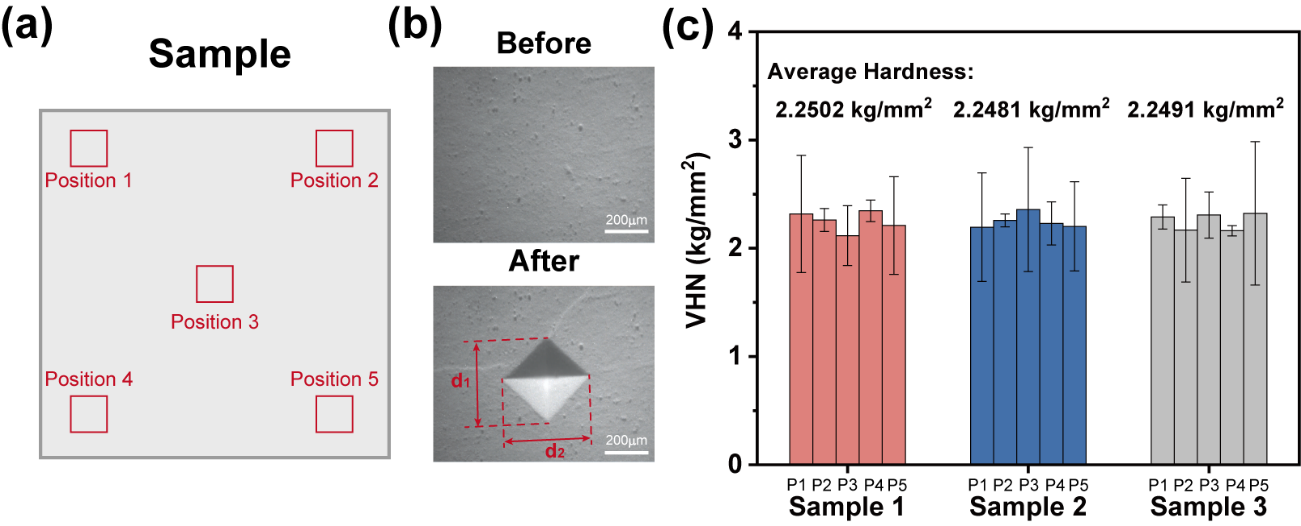


Fig. S7. (a) Schematic of different indentation positions selected for hardness tests on each sample; (b) Optical images of an indentation on bi-layer meta-composite; (c) *VHN* results of different positions on 3 samples.


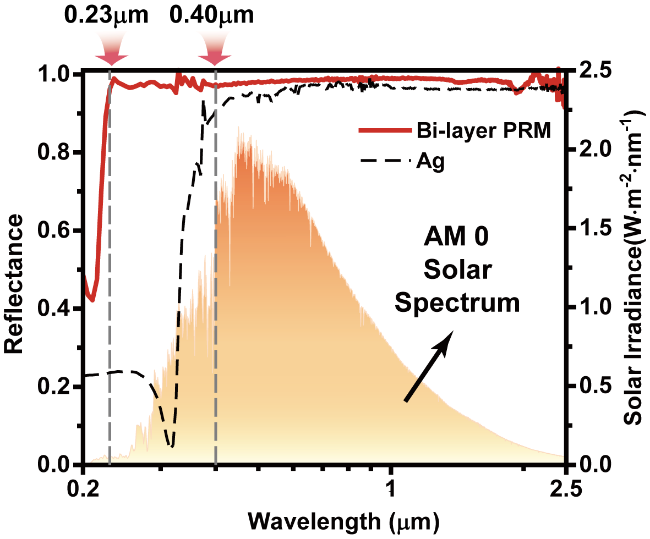


Fig. S8. Experimental reflectance spectra of the bi-layer PRM meta-composite and the silver reflector.


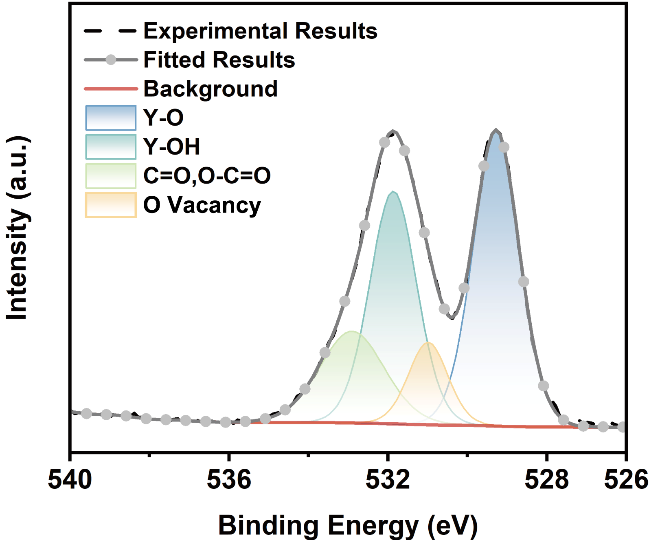


Fig. S9. XPS spectrum of O 1s for the pristine bi-layer photonic random media meta-composite. The peak with the binding energy of ~529.4 eV represents the lattice oxygen of Y_2_O_3_. The peak at ~531.7 eV could be related to the hydroxyl groups. And the peak with the binding energy of ~533.0 eV represents the C=O, O-C=O bonds.^15^


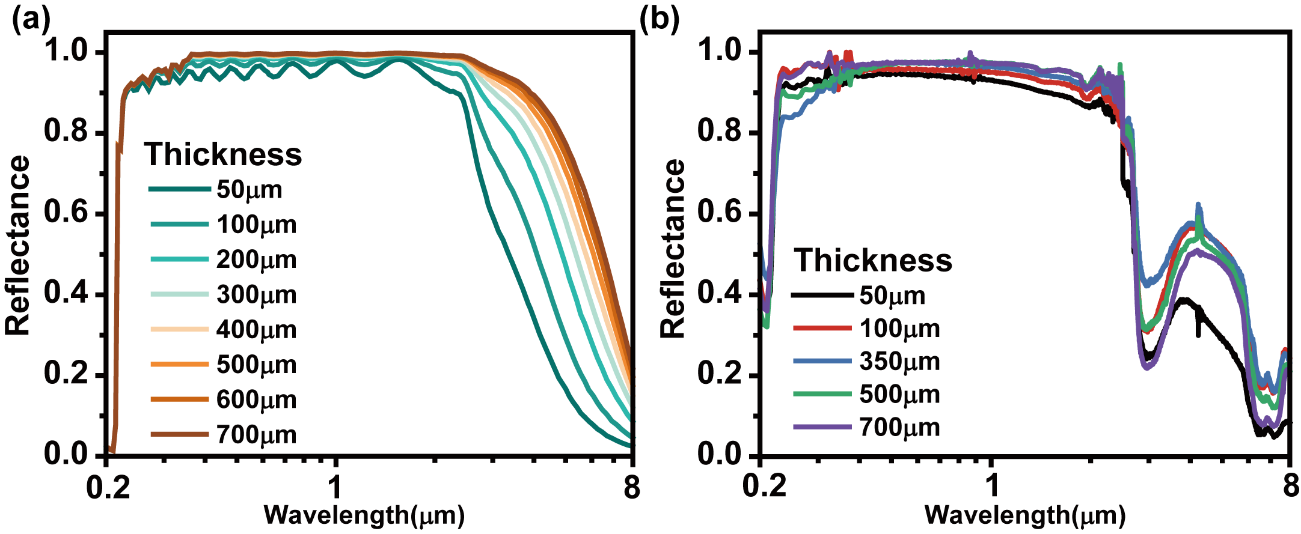


Fig. S10. Calculated reflectance spectrum by the double optimized MC method (a) and the experimental reflectance spectrum (b) of standalone PRM composed of small-scale particles with different thickness.


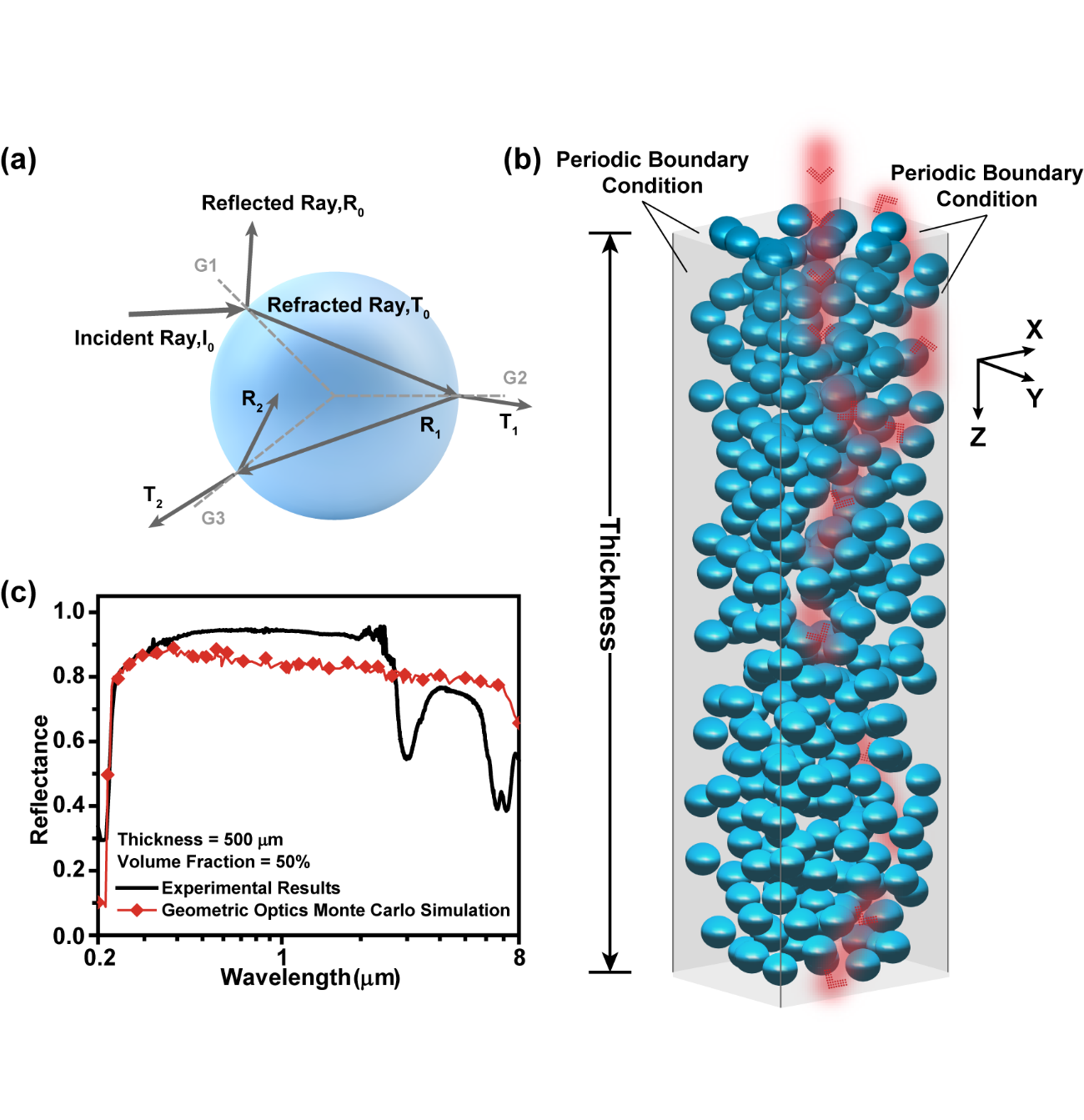


Fig. S11. (a) Schematic image of geometric scattering regime; (b) Schematic image of calculation model for geometric optics MC simulations; (c) Comparison of the reflectance spectrum calculated by the geometric optics Monte Carlo simulation and experimental results.


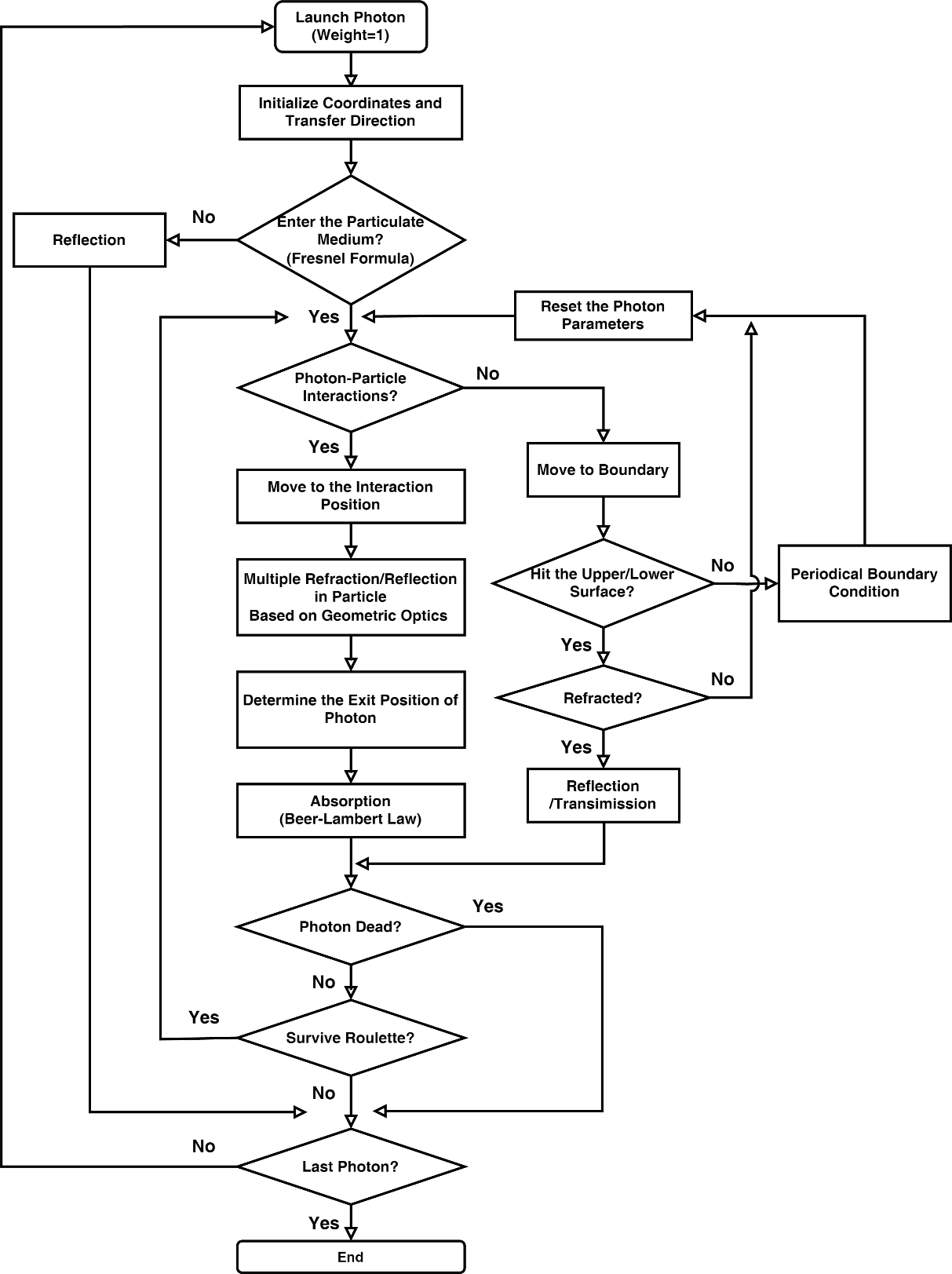


Fig. S12. The flowchart of the geometric optics MC simulation.


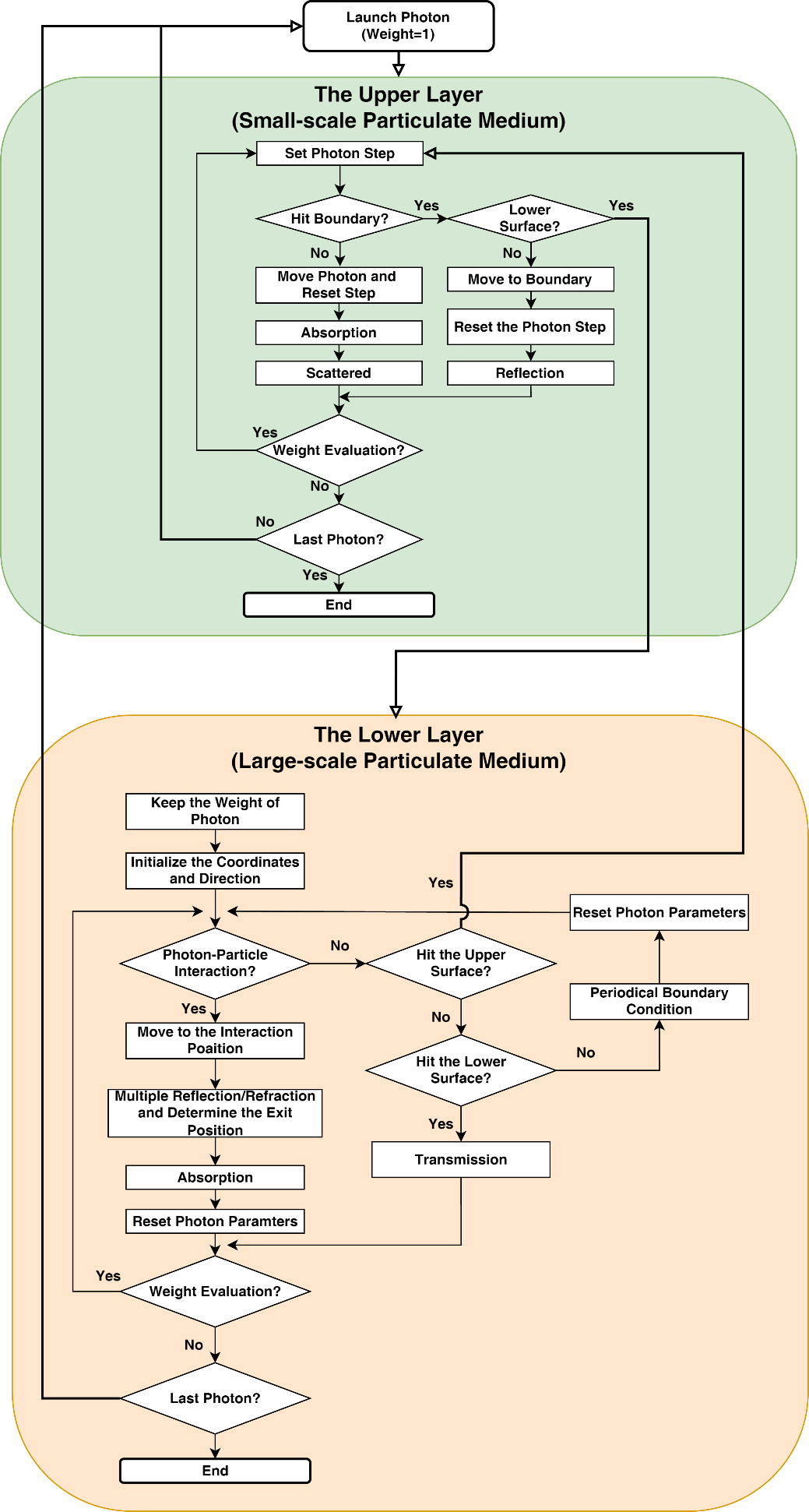


Fig. S13. The flowchart of the continuum calculation model combining the double optimized MC simulation and the geometric optics MC simulation.


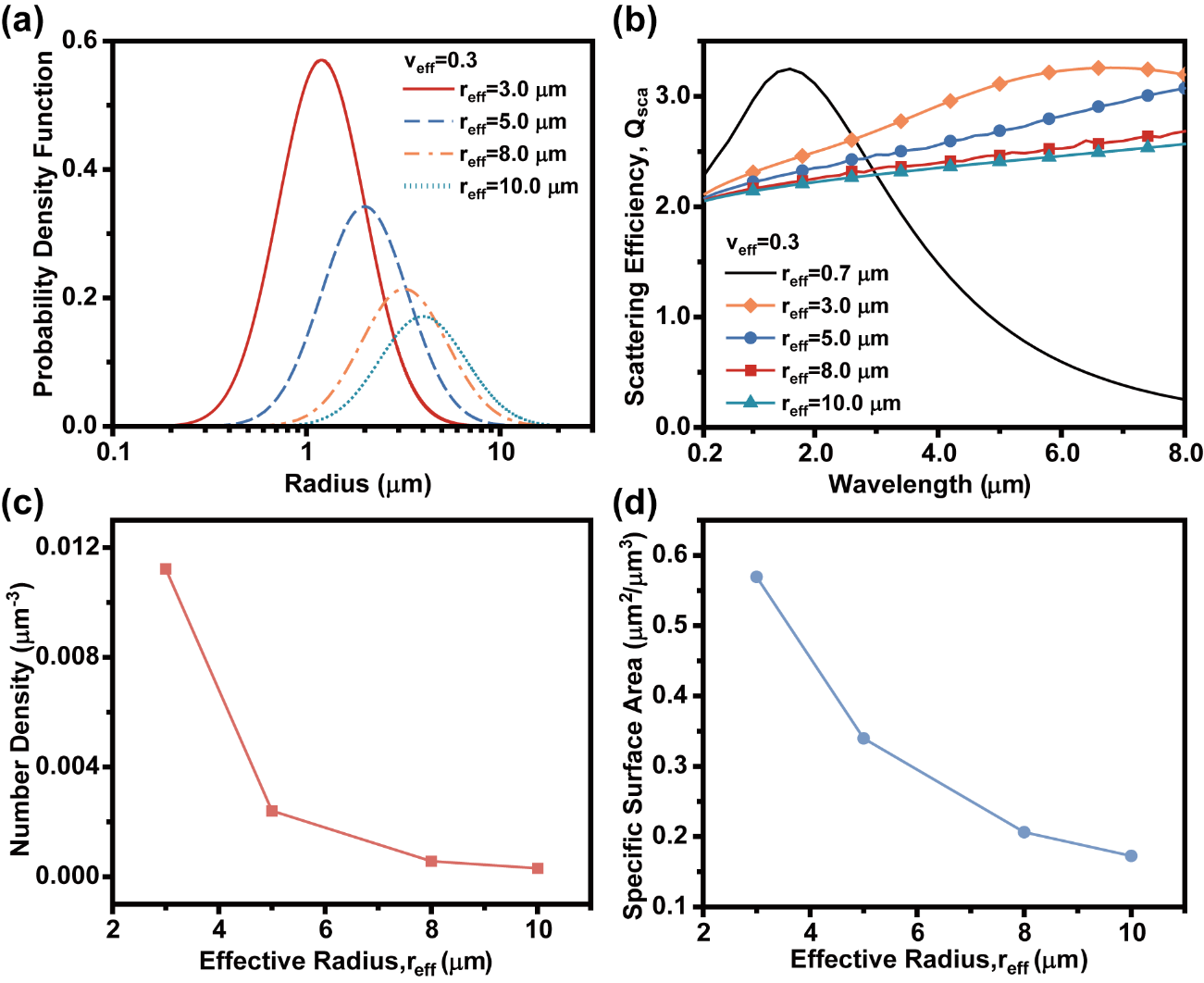


Fig. S14. (a) Probability density function of particles with different size distributions described by the effective radius, *r*_eff_, and effective variance, *v*_eff_. (b) Scattering efficiencies, *Q*_sca_, of particles with different size distributions. Number densities (c) and specific surface area (d) of polydisperse particles with various *r*_eff_ and *v*_eff_ = 0.3.


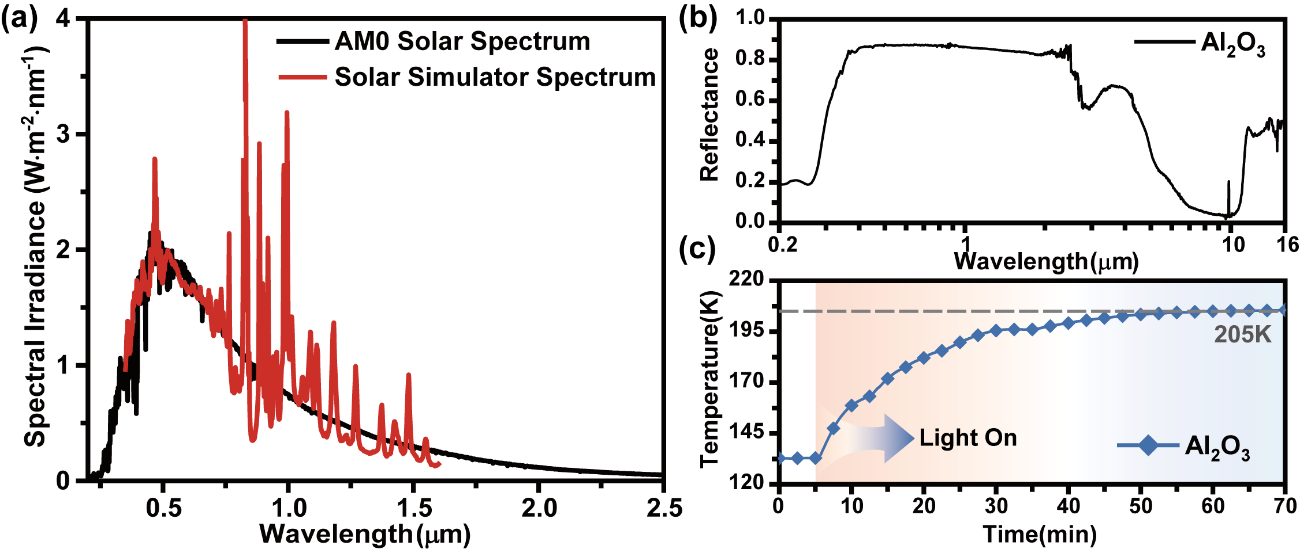


Fig. S15. (a) The irradiance spectrum of AM0 solar simulator and the standard AM0 irradiance spectrum. (b) The experimental reflectance of the dense Al_2_O_3_ ceramic with the thickness of ~500 μm; (c) The dense Al_2_O_3_ ceramic temperature in the home-built space simulator with the AM0 solar simulator on. The equilibrium temperature of the Al_2_O_3_ ceramic was 205 K.


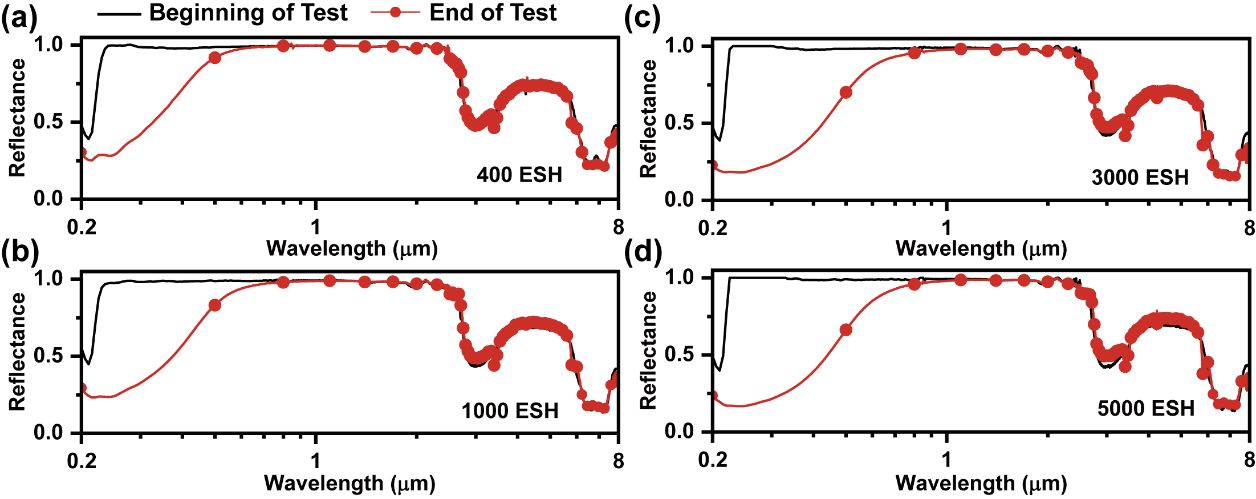


Fig. S16. Reflectance spectra of the bi-layer meta-composite before and after ground-based UV irradiation under different exposure times: (a) 400 ESH, (b) 1000 ESH, (c) 3000 ESH and (d) 5000 ESH.


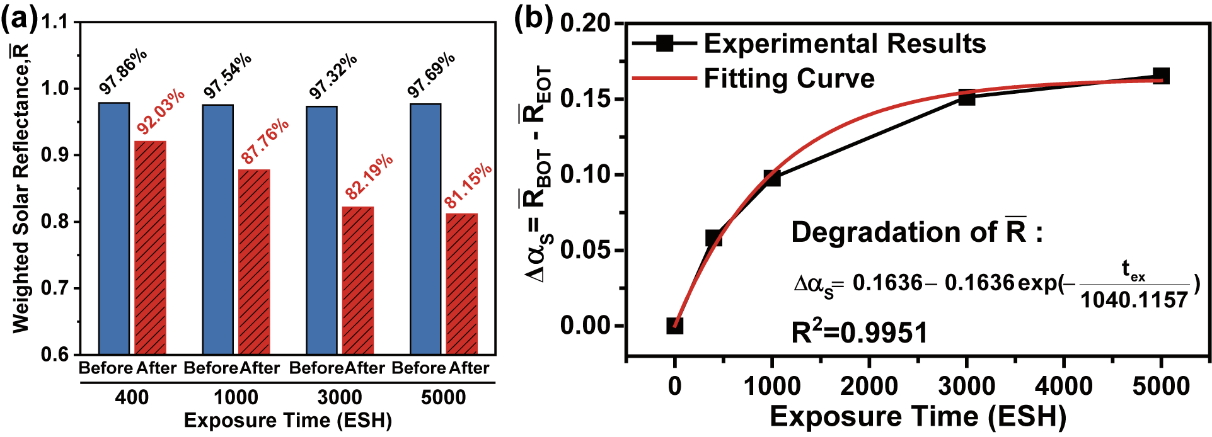


Fig. S17. (a) Weighted solar reflectance ($\bar{\text{R}}$) of samples before and after UV irradiation under different exposure times; (b) The degradation of $\bar{\text{R}}$ induced by UV irradiation and the fitting curve.

To verify the long-duration of the bi-layer meta-composite, we have conducted a series of the ground-based UV irradiation tests. The exposure times of different samples were set as 400 ESH, 1000 ESH, 3000 ESH and 5000 ESH. The reflectance spectra of these samples before and after UV irradiation are compared and displayed in **Fig. S16**. The weighted solar reflectance ($\bar{\text{R}}$) of samples before and after UV irradiation can be calculated and compared in **Fig. S17(a)**. The average $\bar{\text{R}}$ of samples before irradiation tests is 97.60%. And the degradation of $\bar{\text{R}}$, which is represented by the solar absorptance variation (Δ*α*_s_), is fitted by the red curve shown in **Fig. S17(b)**. The fitting equation is expressed as

$\Delta\alpha_{\text{s}}=0.1636-0.1636\exp\left(-\frac{t_{\text{ex}}}{1040.1157}\right)$ (S9)

where *t*_ex_ represents the exposure time. The coefficient $R^{2}=0.9951$ demonstrates the goodness of this fit. According to the fitting result, it can be seen that the degradation of $\bar{\text{R}}$ induced by the UV irradiation almost reached stability when the exposure time longer than 3000 ESH. The $\bar{\text{R}}$ of samples after 3000 and 5000 ESH UV irradiation is 82.19% and 81.15%. This feature of degradation saturation demonstrates that the reflectance of bi-layer meta-composite will not undergo further significant degradation even under constant ultraviolet irradiation during long-duration space missions. Moreover, the Equation (S9) provides a reliable foundation for estimating the lifetime of our meta-composite and predicting long-term on-orbit temperature variations during space missions.


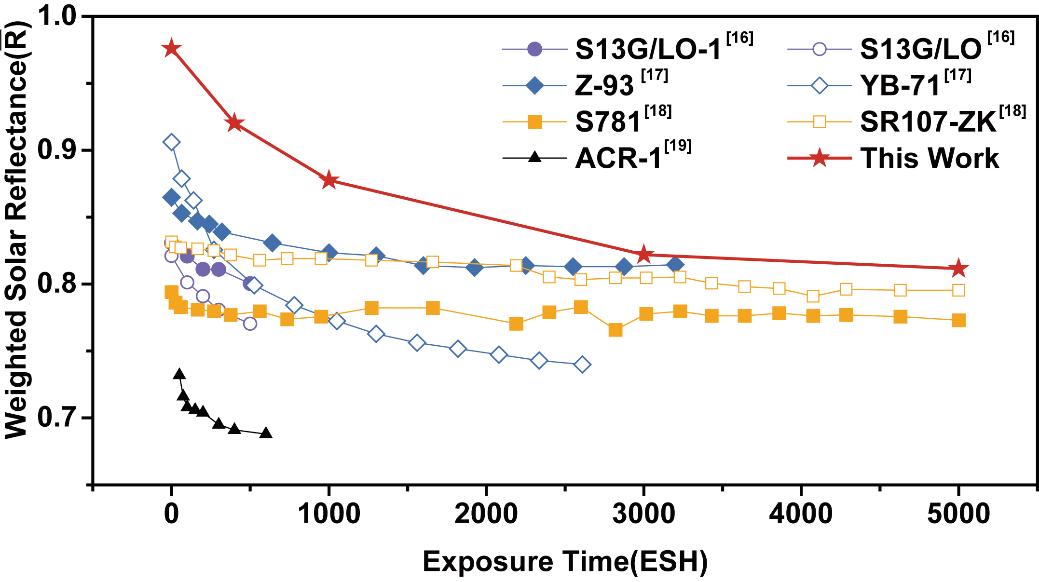


Fig. S18. Comparison of reflectance degradation between the bi-layer meta-composite and existing thermal control coatings under long-term UV irradiation.

The UV irradiation results of bi-layer meta-composite were compared with those of existing thermal control coatings based on particulate medium structures^16-19^ (**Fig. S18**). Before UV exposure, the bi-layer meta-composite exhibited a significantly higher initial $\bar{\text{R}}$ (~97.60%) compared to other coatings. The weighted reflectance of all coatings degraded during UV irradiation. And this degradation trended toward saturation with prolonged exposure time. The comparison of $\bar{\text{R}}$ degradation curves demonstrates that our meta-composite consistently maintained a higher weighted reflectance than other existing coatings throughout the UV irradiation period. This indicates that the meta-composite can sustain superior space thermal control performance under long-term UV irradiation compared to existing coatings, suggesting its full suitability for long-duration space missions.


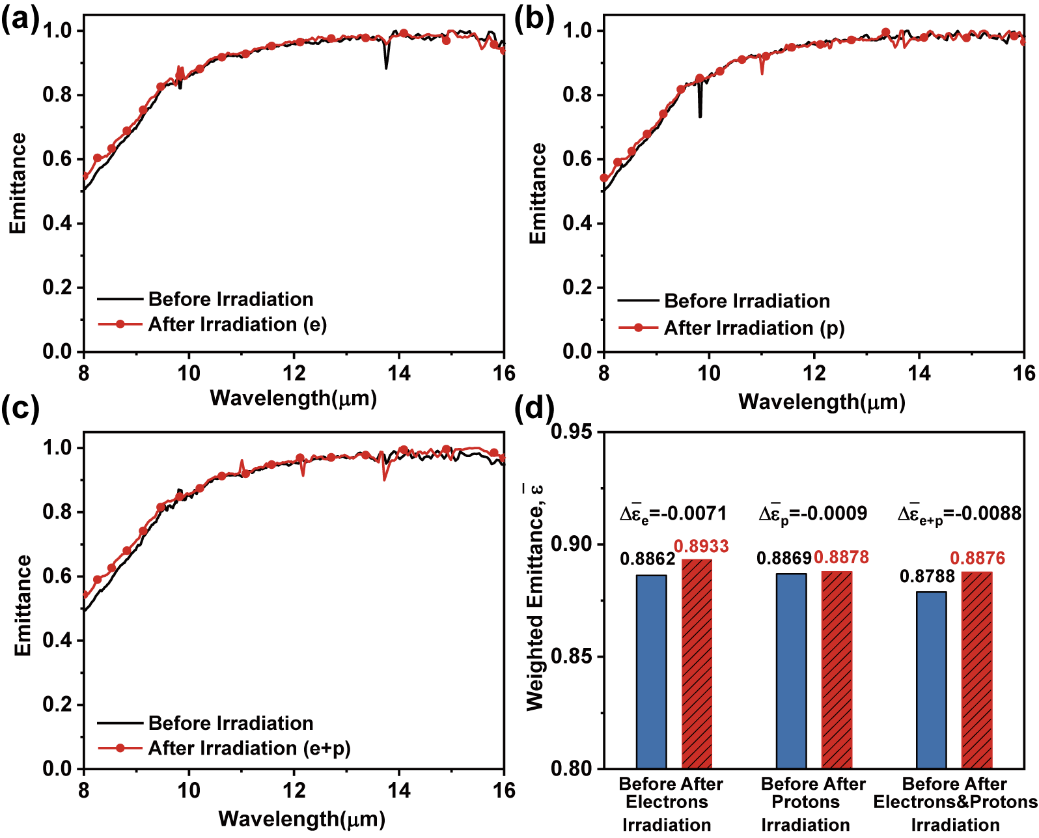


Fig. S19. The emittance spectra comparisons between pristine samples and ones after electrons irradiation (a), protons irradiation (b) and electrons & protons irradiation (c); (d) Variations in weighted emittance of samples before and after charged particles irradiation.


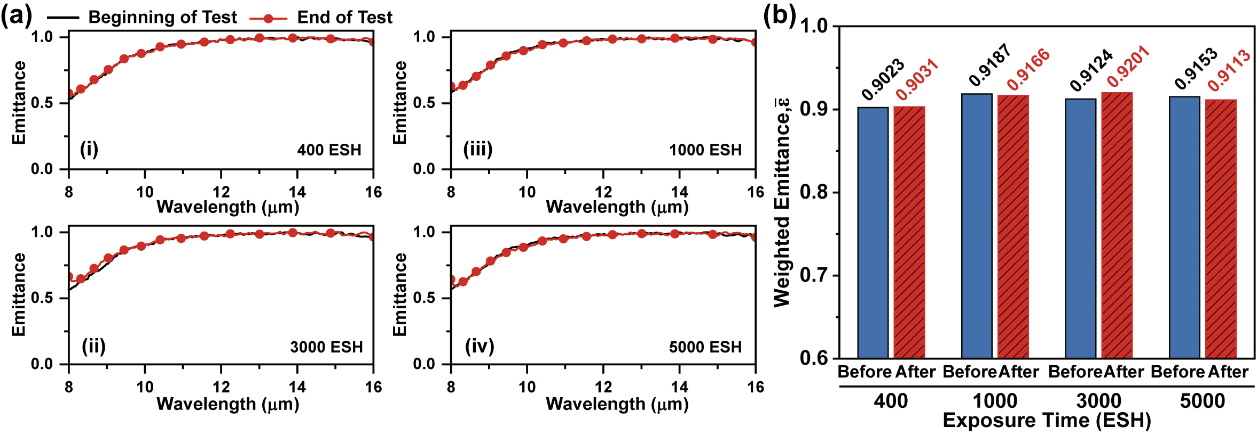


Fig. S20. (a) Emittance spectra and (b) weighted emittance of samples before and after ground-based UV irradiation under different exposure times.


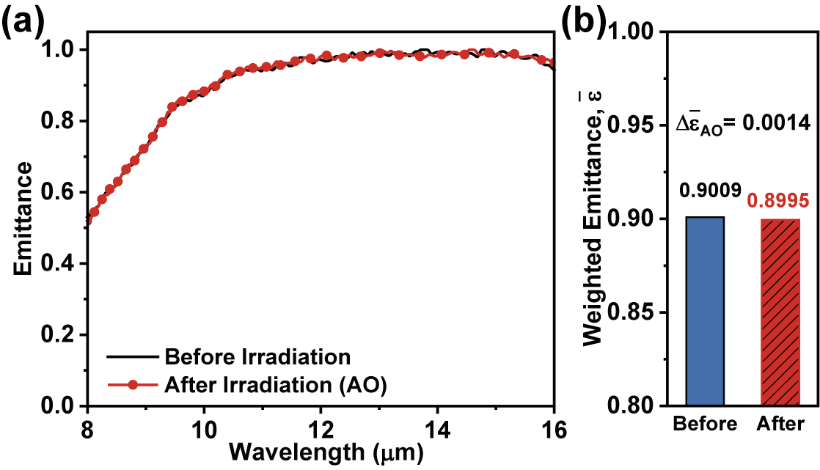


Fig. S21. Emittance spectra comparison (a) and weighted emittance (b) of the sample before and after the atomic oxygen (AO) irradiation test.


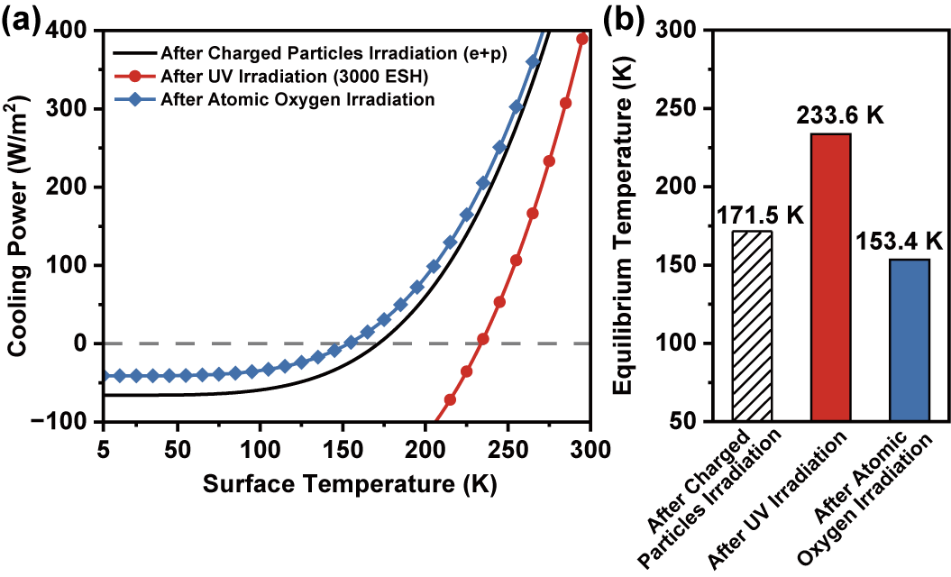


Fig. S22. (a) Relationship between cooling power and temperature of the bi-layer meta-composite after irradiation tests; (b) Equilibrium temperatures of the bi-layer meta-composite after irradiation tests.


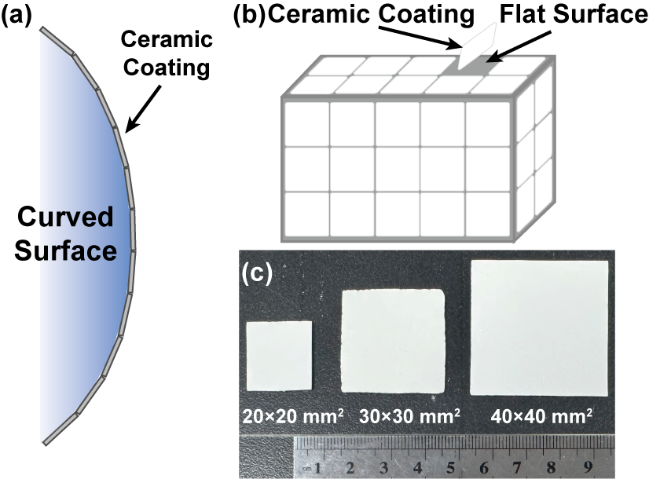


Fig. S23. Schematic of the mounting method for Optical Solar Reflectors (OSR) and our bi-layer meta-composite on the (a) curved surface and (b) flat surface; (c) Bi-layer meta-composite with different sizes.

Aiming to practical large-scale applications, the bi-layer meta-composite can be attached to the outer surfaces of spacecraft by use of an adhesive materials, following the mounting method for Optical Solar Reflectors (OSR)^20,21^. The bi-layer meta-composite can be pre-cut and stored in form of 40 mm × 40 mm square tiles, a typical size of OSR for general use and assembly into large-scale^21-23^ (**Fig. S23(a)** and **(b)**). For complex surfaces, the area, shape, and mounting method can be adjusted to fit various substrates. For large flat spacecraft panels, typical-sized bi-layer meta-composite tiles can be directly attached to substrates using adhesives like silicone resins or epoxy resins (**Fig. S23(b)**). For curved surfaces, typical-sized or small-sized rigid tiles (e.g., 20 mm × 20 mm in **Fig. S23(c)**) can be fabricated and applied in a tiled arrangement (**Fig. S23(a)**). The curvature can be conformed effectively by these small-sized tiles via approximating curves with straight segments method. In areas such as edges or corners where tiles adhesion is not feasible, thermal control white paints can be sprayed as a supplementary coating.


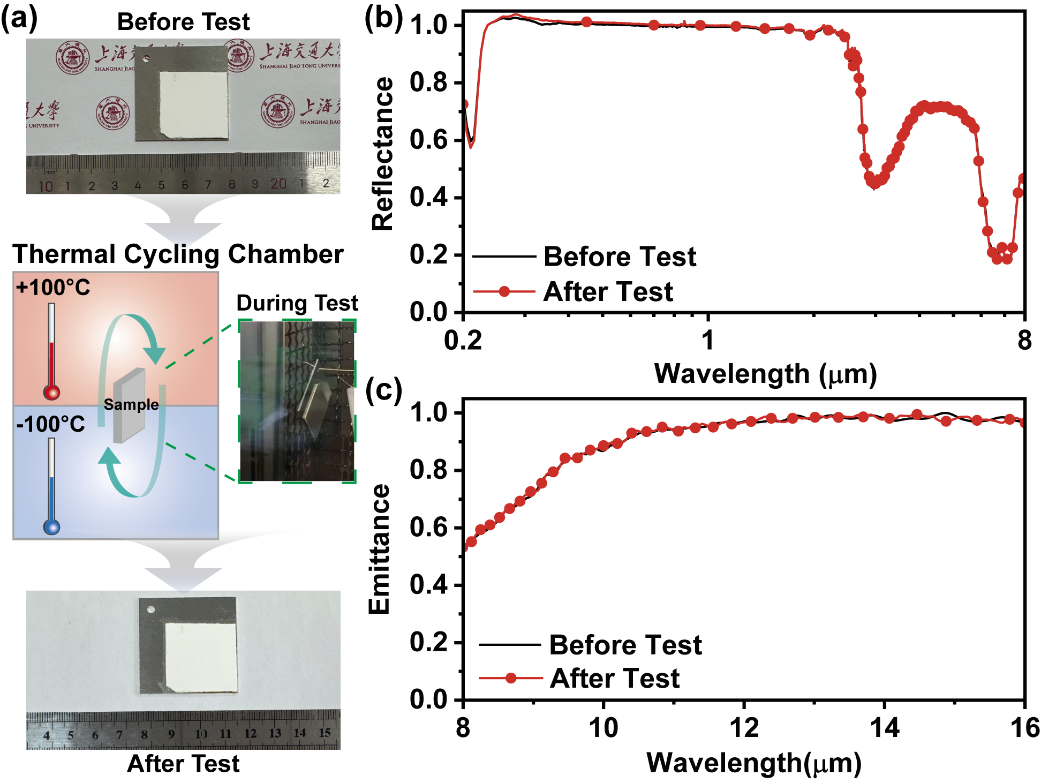


Fig. S24. (a) Images of the sample for the thermal cycling test; (b) Reflectance spectra and (c) emittance spectra of the sample before and after the thermal cycling test.

To verify the mechanical resilience of the bi-layer meta-composite, the thermal cycling test was carried on according to the thermal control coating test standard^24^. The 30 mm × 30 mm square bi-layer meta-composite was attached to an aluminum alloy sheet (**Fig. S24(a)**). Then, about 20 cycles of thermal cycling was tested between -100 ℃ and 100 ℃ with dwell time of 10 min in a thermal cycling chamber. After the thermal cycling test, no mechanical degradation, such as spalling and delamination, was observed (**Fig. S24(a)**). The reflectance and emittance spectra were also remained unchanged (**Fig. S24(b)** and **(c)**).

**Reference**

1. Li, H. C. et al. Prediction of optical properties in particulate media using double optimization of dependent scattering and particle distribution. *Nano Letters* **24**, 287-294 (2024).

2. Hansen, J. E. & Travis, L. D. Light scattering in planetary atmospheres. *Space Science Reviews* **16**, 527-610 (1974).

3. Cartigny, J. D., Yamada, Y. & Tien, C. L. Radiative transfer with dependent scattering by particles: part 1-theoretical investigation. *Journal of Heat Transfer* **108**, 608-613 (1986).

4. Kumar, S. & Tien, C. L. Dependent absorption and extinction of radiation by small particles. *Journal of Heat Transfer* **112**, 178-185 (1990).

5. Singh, B. P. & Kaviany, M. Independent theory versus direct simulation of radiation heat transfer in packed beds. *International Journal of Heat and Mass Transfer* **34**, 2869-2882 (1991).

6. Simonot, L. et al. Ray scattering model for spherical transparent particles. *Journal of the Optical Society of America A* **25**, 1521-1534 (2008).

7. Tsang, L. et al. Scattering of Electromagnetic Waves: Numerical Simulations. 414-418 (New York: John Wiley & Sons, Inc., 2001).

8. Bohren, C. F. & Huffman, D. R. Absorption and Scattering of Light by Small Particles. 102-104 (New York: John Wiley & Sons, Inc., 1998).

9. Meille, S. et al. Mechanical properties of porous ceramics in compression: On the transition between elastic, brittle, and cellular behavior. *Journal of the European Ceramic Society* **32**, 3959-3967 (2012).

10. Yin, Z. J. et al. Evaluating microhardness of plasma sprayed Al_2_O_3_ coatings using Vickers indentation technique. *Journal of Physics D: Applied Physics* **40**, 7090-7096 (2007).

11. Ueno, A., Kim, J. & Nagano, H. Thermophysical properties of metal-insulator transition materials during phase transition for thermal control devices. *International Journal of Heat and Mass Transfer* **166**, 120631 (2021).

12. Qi, H. et al. Studies on atomic oxygen erosion resistance of deposited Mg-alloy coating on Kapton. *Corrosion Science* **124**, 56-62 (2017).

13. Goto, A. et al. Property changes in materials due to atomic oxygen in the low Earth orbit. *CEAS Space Journal* **13**, 415-432 (2021).

14. Yin, J., Li, Y. Y. & Wu, Y. Q. Near-net-shape processed ZnS ceramics by aqueous casting and pressureless sintering. *Ceramics International* **42**, 11504-11508 (2016).

15. Barve, S. A. et al. Effect of argon ion activity on the properties of Y_2_O_3_ thin films deposited by low pressure PACVD. *Applied Surface Science* **257**, 215-221 (2010).

16. Harada, Y., Mell, R. J. & Wilkes, D. R. Effect of the space environment on thermal control coatings. *SPIE Proceedings* **1330**, 90-101 (1991).

17. Cerbus, C. A. & Carlin, P. S. Evaluation of reformulated thermal control coatings in a simulated space environment. Part 1: YB-71. Eighteenth Space Simulation Conference: Space Mission Success Through Testing. Washington, D.C.: NASA, 1994.

18. Feng, W. Q. et al. Combined low-energy environment stimulation test of geosynchronous satellite thermal control coatings. *Journal of Spacecraft and Rockets* **46**, 11-14 (2009).

19. Shen, Z. C. et al. Effect of uncertainty of ground simulation test on the thermal performance of ACR-1 white paint under NUV irradiation. 2018 12th International Conference on Reliability, Maintainability, and Safety (ICRMS). Shanghai, China: IEEE, 2018.

20. Amore, L. J. & Saylor, W. P. Optical solar reflector and mounting method. US patent 5400986 (1995).

21. Sahin, U., Atilgan, Y. A. & Kumbasar, O. M. Inspectability and POD investigation for optical solar reflector bonded satellite panels. *e-Journal of Nondestructive Testing* **28** (2023).

22. Moser, M., Ranzenberger, C. & Duzellier, S. Semi-automated bonding of OSR – qualification of a novel process. 46th International Conference on Environmental Systems. Vienne, Austria: HAL, 2016.

23. Asainov, O. K. et al. Magnetron sputtering in rigid optical solar reflectors production. *Journal of Physics: Conference Series* **729**, 012012 (2016).

24. Lv, J. P., Xie, J. D. & Vitaly, N. Development of Zn_2_SiO_4_ and hexagonal BN inorganic thermal-control coatings with novel thermophysical property. *International Journal of Heat and Mass Transfer* **218**, 124791 (2024).
